# Evaluation of coatings for application in raffia big bags in conditioned storage of soybean cultivars in seed processing units

**DOI:** 10.1371/journal.pone.0242522

**Published:** 2020-11-19

**Authors:** Paulo Carteri Coradi, Roney Eloy Lima, Charline Zaratin Alves, Paulo Eduardo Teodoro, Ana Carina da Silva Cândido

**Affiliations:** 1 Campus Cachoeira do Sul, Federal University of Santa Maria, Cachoeira do Sul, RS, Brazil; 2 Campus de Chapadão do Sul, Federal University of Mato Grosso do Sul, Chapadão do Sul, MS, Brazil; 3 Department of Agricultural Engineering, Federal University of Santa Maria, Santa Maria, RS, Brazil; Indian Institute of Food Processing Technology (IIFPT), INDIA

## Abstract

Different regions have different environmental conditions, which may be unfavorable for the preservation of the quality of stored soybean seeds over time. Thus, it is necessary to adopt specific technologies to control the storage environment conditions. Big raffia bags are widely used for the storage of soybean seeds, however these consist of a porous, permeable material that allows the exchange of gases between the packaging and the storage environment. In an effort to find a solution to this problem, in this study we evaluated low cost big bag coating alternatives, in order to minimize the effects of temperature and intergranular humidity on stored seeds. Thus, the aim of this work was to evaluate the quality of soybean cultivars subjected to different temperature and storage duration conditions and stored in raffia bags with or without internal coating. We used a completely randomized, three-factor (10 × 6 × 5) experimental design. We assessed 10 soybean cultivars, six storage environments, and five evaluation periods. Our results showed that seeds of the M-SOY 8866, M7110 IPRO, CD 2737 RR, and BMX DESAFIO 8473 RSF soybean cultivars preserved their physiological quality better in different storage environments. The storage duration had a cumulative effect on the negative factors that favor the deterioration of the quality of the stored seeds. The storage temperature was the main factor that affected the physiological quality of the stored seeds. The use of coated packaging was beneficial in preserving the physiological quality of stored soybean seeds; however, its effect was greater at ambient temperature than in a cold environment. The best storage environment for the preservation of the quality of the seeds was characterized by 10°C temperature conditions and the use of coated packaging, while the worst storage environment was characterized by ambient temperature conditions without the use of coated packaging. Thus, it was concluded that the use of coatings in raffia big bags can be an alternative for maintaining the quality of seeds of different soybean cultivars during storage in seed processing units.

## 1. Introduction

The expansion of soybean cultivation in the world necessitates the improvement of agricultural production processes with the use of precision technologies, which are associated with genetic improvements and seed conservation, thereby creating the need for the production of seeds with better physiological and sanitary qualities [1]. Among other factors, the physiological quality of the seeds depends on production management and the conduct of post-harvest operations [2].

Storage is an important post-harvest stage, the main objective of which is to conserve seed quality by reducing the speed and intensity of the deterioration process as much as possible [2–5]. When storage conditions are not adequate, soybean seeds suffer viability losses owing to the increased metabolic activity that promotes a reduction in their physiological quality [6,7]. According to Zuchi et al. [8], some soy producing areas are located in regions with tropical climate and high average temperatures, which are considered unfavorable for the preservation of the physiological quality of soybean seeds. Under these conditions, cooling the seeds can contribute to the preservation of stored seed quality.

Artificial cooling is a technique efficient in reducing metabolic activity and, consequently, preserving seed quality in storage [9,10]. This process can take place either during bagging or during the storage period [11]. Mbofung et al. [12] verified the negative effects of ambient temperature on seed deterioration during storage compared to refrigerated environments.

Another factor that interferes with physiological quality is the packaging material used for seed storage. On a commercial scale, soybean seeds are stored and transported from the processing units to rural producers in big, semi-permeable raffia bags. According to Santos et al. [13], the storage of seeds in permeable packaging without water content control facilitates the exchange of humidity with the environment. In turn, this causes an increase in the water content owing to the alteration of the hygroscopic balance of the seeds during storage, which deteriorate more and have reduced vigor and batch viability.

The storage duration of the seeds can intensify their deterioration; one of the techniques employed to minimize this problem is the artificial cooling of the seed mass [14]. Zuchi et al. [8] observed that artificially cooled stored seed lots preserved their physiological quality compared to uncooled stored seeds. According to Ferreira et al. [15], storing seeds at low temperatures reduces their metabolism and the chances of attack by pathogenic microorganisms, thereby preserving the vigor and viability of seed germination.

In view of the unfavorable climatic diversities that occur when it comes to storing soybean seeds in different producing regions, this work aimed to evaluate the effect of temperature and storage duration on the quality and physical and physiological characteristics of different soybean seed cultivars. To this end, we used raffia packaging, which was either coated or not coated with polyethylene material.

## 2. Material and methods

### 2.1 Characterization of the experiments

The research work was carried out at the Seed Laboratory of the Federal University of Mato Grosso do Sul (UFMS), Chapadão do Sul Campus (CPCS), in partnership with the Post-Harvest Laboratory (LAPOS) of the Federal University of Santa Maria (UFSM).

We used a completely randomized, three-factor (10 × 6 × 5) experiment experimental design. We assessed 10 soybean cultivars: (CD 2737 RR, BMX FLECHA 6266 RSF, NS 7209 IPRO, BMX FOCO 74I77 RSF IPRO, DM 75I76 RSF IPRO, ST 797 IPRO, BMX CHALLENGE 8473 RSF, BMX BONUS 8579 RSF IPRO, M7110 IPRO, and M-SOY 8866) and six storage environments (ambient temperature in the raffia bag, ambient temperature in the polyethylene coated raffia bag, cooled to 15°C in the raffia bag, cooled to 15°C in the polyethylene coated raffia bag, cooled to 10°C in the raffia bag, and cooled to 10°C in the polyethylene coated raffia bag) at five evaluation time points (0, 3, 6, 9, and 12 months). Every three months, three packages (i.e., three repetitions) of each treatment were sampled to make quality assessments. After this procedure, the packaging was discarded.

The raffia bags were made of 20 cm (wide) x 30 cm (height) x 0.25 cm polypropylene material. The polyethylene coating used to store the seeds in the raffia bags had dimensions of 20 cm (wide) x 30 cm (height) x 0.1 cm (thick of high density) being produced by the company specialized in food packaging (Videplast Company). The polyethylene packages were constituted by partially crystalline and flexible thermoplastic resin material obtained through the ethylene polymerization, having low density, high tenacity, good impact resistance, flexibility, easy processability, electrical properties and stability, and low permeability to water. It is formed by polar organic compounds and can be changed by temperature environment.

To assess the effects of the storage environments on the physical and physiological quality of the soybean seeds, the three conditions (packaging, temperatures conditions, and storage time) were grouped to define the storage environments (Table 1).

**Table 1:** Experimental design and grouping of storage environments.

Packaging	Temperature Storage (°C)	Storage time (months)	Environments
With coating	Ambient (range 20 to 30°C)	0	E1
With coating	Ambient (range 20 to 30°C)	3	E2
With coating	Ambient (range 20 to 30°C)	6	E3
With coating	Ambient (range 20 to 30°C)	9	E4
With coating	Ambient (range 20 to 30°C)	12	E5
With coating	15	0	E6
With coating	15	3	E7
With coating	15	6	E8
With coating	15	9	E9
With coating	15	12	E10
With coating	10	0	E11
With coating	10	3	E12
With coating	10	6	E13
With coating	10	9	E14
With coating	10	12	E15
Uncoating	Ambient (range 20 to 30°C)	0	E16
Uncoating	Ambient (range 20 to 30°C)	3	E17
Uncoating	Ambient (range 20 to 30°C)	6	E18
Uncoating	Ambient (range 20 to 30°C)	9	E19
Uncoating	Ambient (range 20 to 30°C)	12	E20
Uncoating	15	0	E21
Uncoating	15	3	E22
Uncoating	15	6	E23
Uncoating	15	9	E24
Uncoating	15	12	E25
Uncoating	10	0	E26
Uncoating	10	3	E27
Uncoating	10	6	E28
Uncoating	10	9	E29
Uncoating	10	12	E30

The agronomic characteristics of the soybean cultivars are shown in Table 2.

**Table 2:** Cultivars and their main characteristics.

Cultivars	Cycle (days)	Maturity group	Productivity (bags/hectare)
CD2737RR (1)	127–132	7.3	69.5
BMX FLECHA 6266 RSF IPRO (2)	100–112	6.6	106.3
NS 7209 IPRO (3)	105–115	7.3	81.8
BMX FOCO 74I77 RSF IPRO (4)	108–114	7.2	88
DM 75I76 RSF IPRO (5)	100–113	7.5	90.5
ST 797 IPRO (6)	118–120	7.9	58.3
BMX CHALLENGE 8473 RSF (7)	105–114	7.4	94.7
BMX BÔNUS 8579 RSF IPRO (8)	118–120	7.9	93
M7110 IPRO (9)	102–112	6.2	77
M-SOY 8866 (10)	125–130	8.8	76

### 2.2 Sampling and quality analysis of soybean seeds

The soybean seeds were obtained from the production fields of a rural property in the municipality of Chapadão do Céu-GO and were cleaned to remove impurities and foreign matter (LC 160 machine, Kepler Weber). Then, they were dried in drying silos with radial airflow (Rome Silos Company). The dryer is built in modulated wooden panels (2.11 m x 0.60 m) with treated boards interspersed with aluminum shutters, fixed by galvanized wire and structured with laminated angle arches, mounted overlapping on a self-draining metallic background. Radial ventilation through central tube and centrifugal fan. The temperature of the seed drying air, up to 12% (w.b.) of water content, was 40°C. Then, the seeds were processed using spiral equipment (brand Rota, model Rota II) and a dissymmetric table (brand Silomax, model SDS-80), in order to standardize their size and weight. The seed lots were stored in raffia bags (polypropylene) in air-conditioned warehouses with temperature control. Nine-kilogram seed samples were collected from the bags containing each cultivar, with the aid of a manual presser (EAGRI Equipments), in order to be stored experimentally in different storage environments.

During the storage period, the temperature of the seed mass was monitored weekly with the aid of a digital thermohygrometer (Novus^®^, model Logbox-RHT-LCD), and every three months, the seed samples were collected for quality assessment. The water content of the seeds was determined in a forced air circulation oven (220 L, Tecnal Company) at 105°C ± 1°C, for 24 h, with four repetitions. Then, the samples were removed and placed in a desiccator for cooling (5 L, Tecnal Company) and subsequent weighing (Shimadzu, model B13200H), according to the recommendations of the Rule for Seed Analysis [16]. The water content was determined by the mass difference of the initial and the final sample, and the results were expressed as a percentage (w.b.). The apparent specific mass of the seeds was determined with the aid of a 150 mL beaker and a precision scale, using the mass/volume ratio, with four repetitions [16].

The electrical conductivity evaluation was carried out with four sub-samples, each containing 25 seeds per experimental unit, weighed on a precision scale of 0.001 g, and placed in plastic cups with 75 mL of distilled water, and was undertaken in a incubator at 25°C, for 24 h. After imbibition, the electrical conductivity of the immersion solution was obtained with the aid of a digital conductivity meter (Digimed CD-21) and the results were expressed in μS cm^-^^1^ g^-^^1^ according to the methodology proposed by Brasil [16].

For the germination test, four sub-samples of 50 seeds from each experimental unit were used, distributed in paper towel rolls (Germitest), and moistened with distilled water in an amount that was 2.5 times the dry paper mass. Then, the rolls with the seeds were placed in a germinator (Mangesdorf), set at a temperature of 25°C ± 2°C. The evaluations were carried out on the fifth and eighth days after the test was installed, by counting normal and abnormal seedlings as well as dead seeds, according to the criteria established in the Rules for Seed Analysis [16].

In the tetrazolium test, four sub-samples of 50 seeds from each experimental unit were used. These were pre-moistened on Germitest paper for 16 h at 25°C and then immersed in a 0.075% tetrazolium solution, in which they were kept for 3 h at 35°C in the dark. After this period, the seeds were washed in running water and their vigor, viability, and moisture damage [6–8] were evaluated according to the methodology established by França-Neto [17].

### 2.3 Statistical analysis

We used analysis of variance, and the treatments and significant interactions were analyzed by the Scott-Knott average test at 5% probability with SK.nest package of R software. Subsequently, data values (storage time and cultivars) were pooled out and compare for each packaging and temperature storage environments for linear regression analysis. We built two heatmap using an average Euclidean distance. The first to demonstrate the differences between storage environments and the second to demonstrate the differences between soybean cultivars. Principal component analysis was also performed to verify the similarity between storage environments and soybean cultivars with ellipses with confidence interval for groups. These analyzes were performed with R software.

## 3. Results

### 3.1 Temperature of stored seed mass

**Fig 1:**
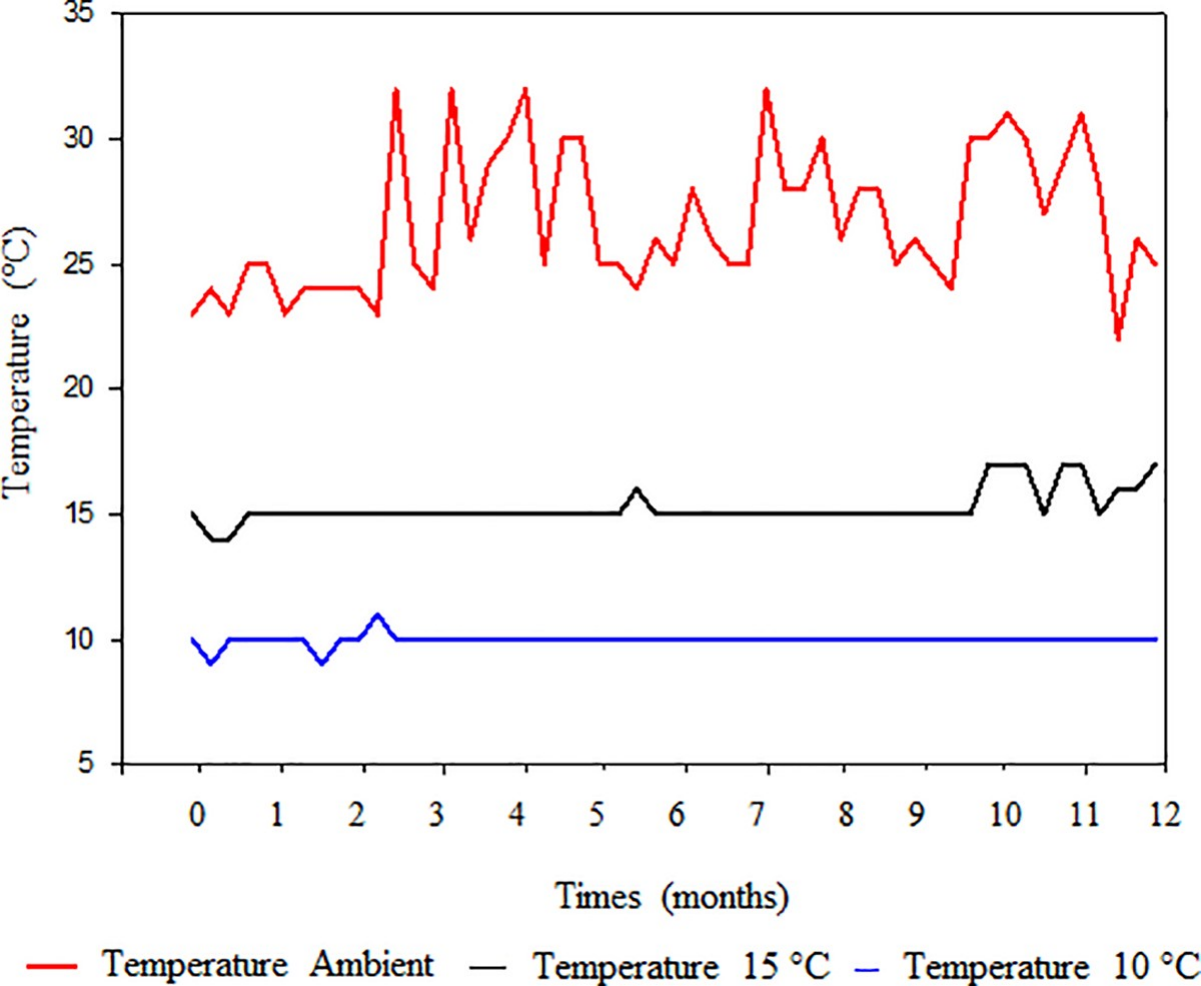
Temperature of seed mass in storage environments.

Under ambient conditions, we observed that the temperature of the stored soybean mass increased, and had greater variation than that of the soybean seed mass stored at 10°C and 15°C (Fig 1). For soybean seeds stored at ambient temperature, the average temperature was 26.7°C, the maximum and minimum temperatures were 32°C and 22°C, respectively, and the thermal range was 10°C. In the 15°C storage conditions, the average temperature was 15.25°C, the maximum and minimum temperatures were 17°C and 14°C, respectively, and the thermal range was 3°C. In the 10°C storage conditions, the average temperature was 9.98°C, the maximum and minimum temperatures were 11°C and 9°C, respectively, and the thermal range was 2°C.

In the analysis of variance (Table 3), it was found that the treatments and interactions were significant at 5% probability, for all seed quality tests.

**Table 3:** Analysis of variance (Mean Squares) of soybean cultivars in storage environments.

FV	TA	ME	1ªCG	G
A	72.22*	4991.0*	12755.37*	7856.17*
C	1.3*	4153.67*	18609.09*	13677.49*
A x C	0.52*	438.45*	341.87*	274.64*
RES	0.09	363.16	32.44	17.69
CV (%)	2.96	2.78	7.44	4.76
Average	10.36	684.36	76.56	88.41
FV	CE	VG TZ	VB TZ	DU TZ
A	36710.43*	9391.36*	4808.68*	5269.43*
C	85227.55*	9717.86*	1685.65*	692.26*
A x C	596.25*	344.23*	157.09*	161.99*
RES	127.63	16.96	7.45	2.61
CV (%)	10.46	5.46	3.02	32.72
Average	108.05	75.43	90.44	4.94

FV—source of variation. A—environments. C—cultivars. RES—residue. CV (%)—coefficient of variation. TA—water content. ME—apparent specific mass. 1st CG—first germination count. G—germination. CE—electrical conductivity. VG TZ—tetrazolium vigor. VB TZ—tetrazolium viability. DU TZ—tetrazolium moisture damage.

*Significant at 5% probability by the F test.

The results of the linear regression analyses (Table 4) of the seed and soybean quality tests showed that the determination coefficients ranged from 60.13% to 98.98%, while the lines behaved similarly and this was indicative of the seed quality throughout the storage period.

**Table 4:** Regression equations and coefficients of determination.

Analyzes	Coatings	Environments	Equations	R^2^
Water content	WC	Ambient (range 20 to 30°C)	*y* = -0.3171*x* + 11.617	88.15
	WC	15°C	*y* = -0.8189*x* + 12.69	92.12
	WC	10°C	*y* = -0.557*x* + 12.252	92.15
	UC	Ambient (range 20 to 30°C)	*y* = 0.041*x* + 10.532	78.13
	UC	15°C	*y* = -1.1029*x* + 12.486	85.15
	UC	10°C	*y* = -0.4704*x* + 12.261	78.13
Apparent specific mass	WC	Ambient (range 20 to 30°C)	*y* = -1.061*x* + 672.73	60.13
	WC	15°C	*y* = -5.4076*x* + 701.2	69.14
	WC	10°C	*y* = -6.0881*x* + 702.52	71.13
	UC	Ambient (range 20 to 30°C)	*y* = -7.084*x* + 707.62	78.16
	UC	15°C	*y* = -4.8283*x* + 697.84	72.68
	UC	10°C	*y* = -6.096*x* + 702.12	75.43
Germination	WC	Ambient (range 20 to 30°C)	*y* = -9.03*x* + 108.7	83.32
	WC	15°C	*y* = -0.71*x* + 94.16	86.73
	WC	10°C	*y* = 0.03*x* + 93.85	98.13
	UC	Ambient (range 20 to 30°C)	*y* = -11.205*x* + 113.88	82.14
	UC	15°C	*y* = -0.08*x* + 90.41	76.20
	UC	10°C	*y* = 0.3*x* + 93.34	84.54
Electric conductivity	WC	Ambient (range 20 to 30°C)	*y* = 20.215*x* + 61.378	90.21
	WC	15°C	*y* = 6.1505*x* + 81.874	78.92
	WC	10°C	*y* = 2.8927*x* + 84.083	88.76
	UC	Ambient (range 20 to 30°C)	*y* = 31.346*x* + 38.801	79.54
	UC	15°C	*y* = 10.52*x* + 73.576	88.68
	UC	10°C	*y* = 2.6684*x* + 85.218	92.58
Vigor	WC	Ambient (range 20 to 30°C)	*y* = -14.065*x* + 108.37	83.76
	WC	15°C	*y* = -1.515*x* + 85.415	88.74
	WC	10°C	*y* = -0.395*x* + 85.855	91.45
	UC	Ambient (average at 24°C)	*y* = -16.61*x* + 114.68	79.42
	UC	15°C	*y* = -6.27*x* + 95.6	88.93
	UC	10°C	*y* = -0.41*x* + 87.2	92.14
Mechanical damage	WC	Ambient (range 20 to 30°C)	*y* = 0.942*x* - 0.494	96.45
	WC	15°C	*y* = 0.84*x* - 0.38	93.65
	WC	10°C	*y* = 0.64*x* + 0.18	97.89
	UC	Ambient (range 20 to 30°C)	*y* = 0.83*x* - 0.07	91.78
	UC	15°C	*y* = 1.51*x* - 1.63	94.56
	UC	10°C	*y* = 0.55*x* + 0.03	95.67
Damage mechanical vigor test	WC	Ambient (range 20 to 30°C)	*y* = -9.4*x* + 95.98	92.35
	WC	15°C	*y* = 0.04*x* + 81.2	93.21
	WC	10°C	*y* = 2.04*x* + 75.36	96.78
	UC	Ambient (range 20 to 30°C)	*y* = -14.23*x* + 105.85	97.89
	UC	15°C	*y* = -0.96*x* + 81.86	98.98
	UC	10°C	*y* = 2.56*x* + 71	94.61
Mechanical damage viability test	WC	Ambient (range 20 to 30°C)	*y* = -6.27*x* + 104.13	96.54
	WC	15°C	*y* = -0.05*x* + 92.95	96.79
	WC	10°C	*y* = 0.35*x* + 90.87	97.32
	UC	Ambient (range 20 to 30°C)	*y* = -9.63*x* + 111.93	98.69
	UC	15°C	*y* = 0.46*x* + 89.06	95.71
	UC	10°C	*y* = 0.11*x* + 93.63	97.48

WC–With coating, UC–Uncoating.

**Fig 2:**
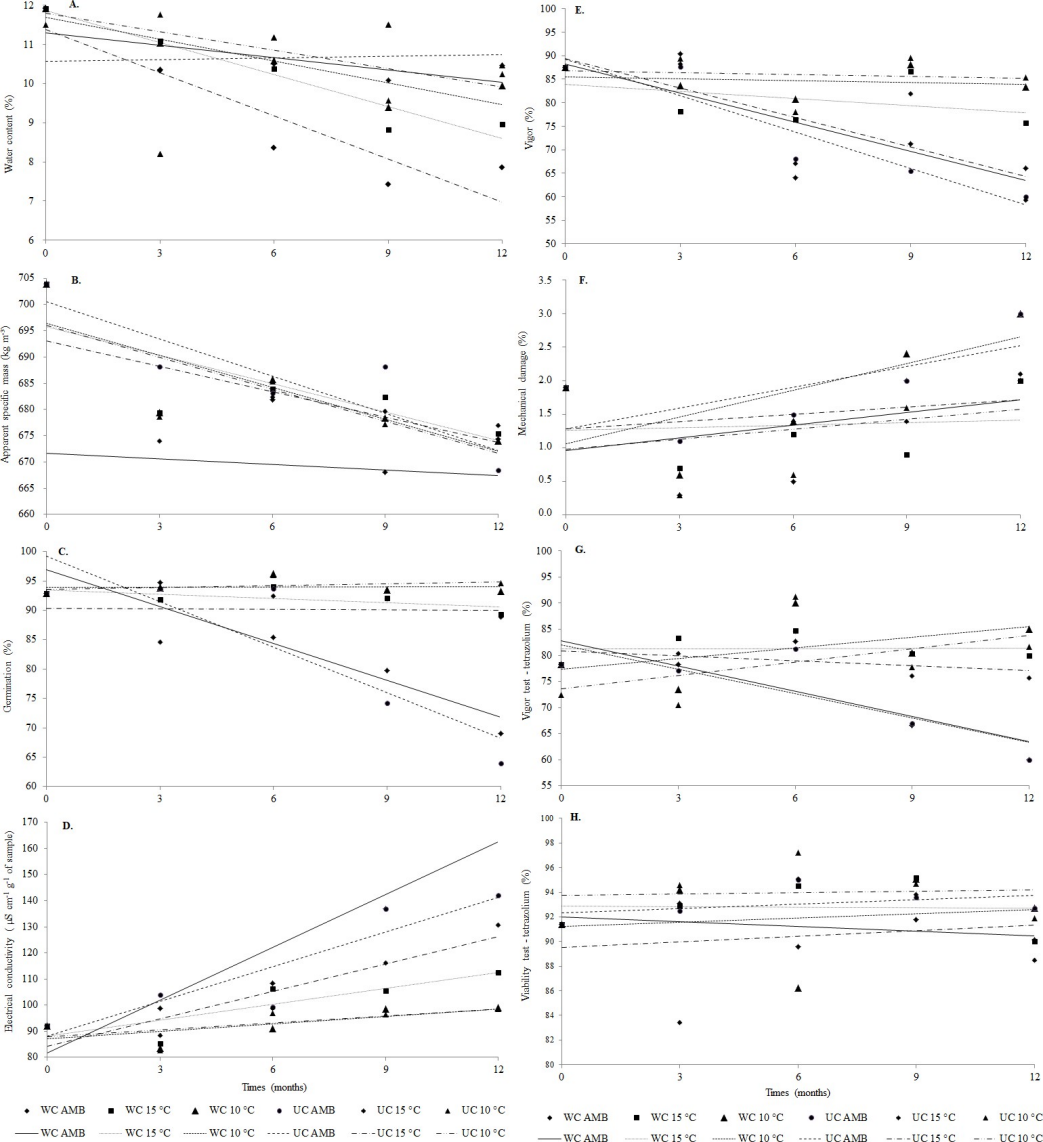
Water content (A), Apparent specific mass (B), Germination (C), Electrical conductivity (D), Vigor (E), Mechanical damage (F), Vigor test–tetrazolium (G), Viability test–tetrazolium (H) of soybean seeds stored in different environments over time. WC–With Coating, UC–Uncoated.

Fig 2 shows the results of the quality analysis of soybean seeds over the storage period.

### 3.2 Water content of stored soybean seeds

According to the results shown in Table 5, the average water contents of the seeds varied depending on the different conditions and storage durations. During the storage period, the water contents of the cultivars ranged from 7% (w.b.) to 13% (w.b.). Our results showed that during storage, the relative humidity influenced the water contents of the hygroscopic materials of the seeds stored in the permeable packaging, which allowed greater water vapor exchanges with the environment. The use of coated packaging preserved a greater balance in terms of the exchange of humidity between the seeds and the storage environment.

**Table 5:** Breakdown of the interaction between storage environments and soybean cultivars for water content (%).

E/Cultivars	1	2	3	4	5	6	7	8	9	10
E1	12.15 aA	11.75 aB	12.10 aA	12.53 aA	11.93 aA	11.77 aB	12.13 aA	12.16 aA	11.04 cC	11.61 aC
E2	10.40 eB	10.79 cA	11.14 cA	10.49 dB	9.85 eC	10.15 dC	10.40 cB	9.76 eC	10.56 cB	10.04 cC
E3	10.61 dA	10.28 cA	9.50 eB	10.60 dA	10.75 cA	10.60 cA	10.61 cA	10.86 cA	10.84 cA	10.32 bA
E4	9.70 fB	10.12 cA	10.05 dB	9.96 eB	10.23 dA	10.63 cA	10.45 cA	10.25 dA	9.68 eB	9.84 cB
E5	10.61 dA	10.32 cB	10.15 dB	10.51 dA	10.57 cA	10.30 dB	10.17 dB	10.56 dA	10.75 cA	10.71 bA
E6	12.15 aA	11.75 aB	12.10 aA	12.53 aA	11.93 aA	11.77 aB	12.13 aA	12.16 aA	11.04 cC	11.61 aB
E7	11.13 cA	11.47 bA	11.30 bA	11.43 bA	10.51 cB	11.48 bA	10.19 dC	11.56 bA	11.07 cA	10.82 bB
E8	10.53 dB	10.48 cB	10.59 dB	8.53 hC	11.25 bA	10.66 cB	10.39 cB	10.47 dB	10.73 cB	10.11 cB
E9	8.84 gA	8.81 eA	8.63 bA	8.97 gA	8.92 fA	8.70 fA	8.79 fA	8.67 fA	9.07 fA	8.79 eA
E10	8.91 gB	9.10 eA	8.83 bB	9.26 fA	8.82 fB	9.11 fA	8.76 fB	8.72 fB	8.90 fB	9.20 dA
E11	12.15 aA	11.75 aB	12.10 aA	12.53 aA	11.93 aA	11.77 aB	12.13 aA	12.16 aA	11.04 cC	11.61 aB
E12	10.76 dB	11.10 bA	11.16 cA	10.51 dB	11.37 bA	11.48 bA	11.26 bA	11.41 bA	10.73 cB	10.64 bB
E13	10.27 eC	10.57 cB	10.24 dC	11.04 cA	10.49 cB	10.78 cA	10.47 cB	11.31 bA	10.06 dC	10.67 bB
E14	9.41 fA	9.49 dA	9.48 eA	9.70 eA	9.54 eA	9.49 eA	9.37 eA	9.67 eA	8.45 gB	9.42 dA
E15	10.16 eA	9.84 dB	10.26 dA	9.98 eA	9.81 eB	10.18 dA	9.61 eB	10.03 dA	9.83 eB	9.82 cB
E16	12.15 aA	11.75 aB	12.10 aA	12.53 aA	11.93 aA	11.77 aB	12.13 aA	12.16 aA	11.04 cC	11.61 aB
E17	8.82 gA	9.06 eA	7.70 gD	8.09 hC	8.16 gC	8.06 gC	7.33 hD	8.12 gC	8.51 gB	8.16 fC
E18	10.26 eC	11.43 Bb	10.93 cB	11.47 bB	11.22 bB	11.30 bB	10.24 dC	11.84 bA	11.43 bB	11.71 aA
E19	10.77 dD	11.48 bC	11.72 bB	11.55 bB	11.59 bB	12.11 aA	11.89 aA	11.56 bB	11.05 cD	11.31 aC
E20	10.64 dA	10.63 cA	10.24 dB	10.45 dA	10.58 cA	10.05 dB	9.97 dB	10.57 dA	10.92 cA	10.66 bA
E21	12.15 aA	11.75 aB	12.10 aA	12.53 aA	11.93 aA	11.77 aB	12.13 aA	12.16 aA	11.04 cC	11.61 aB
E22	10.06 eB	10.53 cA	10.49 dB	10.15 dB	10.11 dB	10.74 cA	10.23 dB	10.33 dB	10.26 dB	10.38 bB
E23	8.04 hB	8.63 eA	8.85 bA	8.41 hA	8.02 gB	8.88 fA	7.87 gB	8.25 gB	8.46 gA	8.19 fB
E24	7.00 jB	7.40 gB	7.53 gB	7.39 iB	7.32 hB	7.92 gA	8.00 gA	7.26 hB	7.37 hB	7.12 gB
E25	7.49 iA	7.93 fA	7.53 gA	7.50 iA	7.00 hB	7.49 hA	7.09 hB	7.19 hB	7.33 hB	11.61 aA
E26	12.15 aA	11.75 aB	12.10 aA	12.53 aA	11.93 aA	11.77 aB	12.13 aA	12.16 aA	11.04 cC	7.47 gB
E27	11.42 bB	12.05 aA	12.00 aA	12.24 aA	11.73 aB	11.65 aB	11.45 bB	11.09 cC	12.02 aA	11.94 aA
E28	11.06 cB	11.50 bA	11.03 cB	11.59 bA	11.54 bA	10.79 cB	10.73 cB	11.34 bA	11.49 bA	10.67 bB
E29	9.57 fA	9.81 dA	9.56 eA	9.45 fA	9.65 eA	9.53 eA	9.29 eA	9.54 eA	9.74 eA	9.55 dA
E30	10.32 eA	10.51 cA	10.06 dA	10.26 dA	10.29 dA	10.23 dA	9.92 dA	10.32 dA	10.23 dA	10.32 bA

Environments (E) according to the schema proposed by Table 1. Cultivars: CD 2737 RR (1), BMX FLECHA 6266 RSF (2), NS 7209 IPRO (3), BMX FOCO 74I77 RSF IPRO (4), DM 75I76 RSF IPRO (5), ST 797 IPRO (6), BMX CHALLENGE 8473 RSF (7), BMX BONUS 8579 RSF IPRO (8), M7110 IPRO (9) and M-SOY 8866 (10). The averages followed by the same lower case letters in the column and upper case letters in the row did not differ statistically at 5% probability by the Scott Knott test. The averages followed by the different lowercase letters in the column and uppercase letters in the row differed statistically at 5% probability by the Scott Knott test.

The increase in the storage duration reduced the water content of the seeds, regardless of packaging or storage temperatures (Fig 2A). The results obtained indicated that coated packaging had a beneficial effect on seed quality, as it allowed for better conservation of the water contents and low heat and mass transfers between the atmospheric air and the seeds in the storage environments. After nine months of storage, the soybean seeds stored at 10°C reached a hygroscopic balance with water levels similar to those of the initial storage conditions. The same pattern was observed in 15°C storage environments. However, in ambient temperature environments, the seeds reached a hygroscopic balance with water contents lower than those achieved in the 15°C and 10°C environment. The air-conditioned environment stabilized the water content of the seeds during storage.

### 3.3 Apparent specific mass of stored soybean seeds

A reduction was observed in the apparent specific mass of the seeds (Table 6), regardless of the storage environment. However, there was no significant difference among the different storage environments in terms of the apparent specific mass (kg m^-^^3^) of the cultivars. Seeds stored in coated packaging and at ambient temperature had the lowest apparent density values, which remained constant over time (Fig 2B).

**Table 6:** Breakdown of the interaction between storage environments and soybean cultivars for the apparent specific mass (kg m^-^^3^).

E/Cultivars	1	2	3	4	5	6	7	8	9	10
E1	698.2 aA	701.4 aA	703.9 aA	685.2 aA	710.3 aA	703.6 aA	704.8 aA	704.9 aA	701.3 aA	724.9 aA
E2	692.5 aA	678.4 bB	689.3 aB	685.0 aB	682.6 bB	676.1 bB	668.0 bB	700.8 aA	704.0 aA	713.0 aA
E3	676.7 aA	676.7 bA	682.3 bA	673.4 aA	674.1 bA	677.8 bA	684.0 bA	682.0 bA	687.7 aA	703.4 bA
E4	690.9 aA	535.6 cB	677.8 bA	671.3 aA	674.5 bA	687.0 bA	687.2 bA	682.8 bA	683.9 aA	689.3 bA
E5	681.8 aA	672.7 bA	662.1 bA	669.2 aA	666.2 bA	670.1 bA	672.2 bA	680.1 bA	688.6 aA	680.7 bA
E6	698.2 aA	701.4 aA	703.9 aA	685.2 aA	710.3 aA	703.6 aA	704.8 aA	704.9 aA	701.3 aA	724.9 aA
E7	683.4 aA	668.9 bA	674.7 bA	664.9 aA	680.6 bA	678.6 bA	671.9 bA	689.5 aA	686.2 aA	694.9 bA
E8	689.4 aA	683.0 bA	676.8 bA	674.4 aA	672.2 bA	664.7 bA	684.2 bA	690.0 aA	703.0 aA	702.0 bA
E9	676.1 aA	682.9 bA	675.0 bA	678.7 aA	675.1 bA	684.2 bA	681.6 bA	684.1 bA	689.3 aA	696.3 bA
E10	674.7 aA	679.0 bA	677.7 bA	669.2 aA	675.8 bA	670.7 bA	671.2 bA	678.1 bA	677.4 aA	679.8 bA
E11	698.2 aA	701.4 aA	703.9 aA	685.2 aA	710.3 aA	703.6 aA	704.8 aA	704.9 aA	701.3 aA	724.9 aA
E12	682.7 aA	672.6 bA	674.6 bA	670.4 aA	684.0 bA	680.6 bA	688.6 bA	672.2 bA	680.8 aA	687.9 bA
E13	692.9 aA	680.0 bA	677.8 bA	679.8 aA	678.7 bA	682.0 bA	670.6 bA	699.2 aA	694.8 aA	701.1 bA
E14	678.0 aA	672.4 bA	674.9 bA	669.6 aA	670.0 bA	684.2 bA	686.7 bA	679.3 bA	678.4 aA	690.1 bA
E15	674.2 aA	670.1 bA	670.8 bA	671.4 aA	671.8 bA	668.9 bA	674.6 bA	677.5 bA	677.7 aA	682.7 bA
E16	698.2 aA	701.4 aA	703.9 aA	685.2 aA	710.3 aA	703.6 aA	704.8 aA	704.9 aA	701.3 aA	724.9 aA
E17	692.4 aA	696.1 aA	688.8 aA	683.4 aA	685.6 bA	685.5 bA	683.5 bA	679.5 bA	687.6 aA	699.6 bA
E18	693.7 aA	686.5 aA	679.1 bA	674.1 aA	683.1 bA	666.8 bA	678.9 bA	693.8 aA	694.8 aA	680.9 bA
E19	681.7 aA	691.8 aA	693.2 aA	684.2 aA	679.7 bA	688.2 bA	684.2 bA	694.4 aA	683.5 aA	700.3 bA
E20	674.4 aA	664.6 bA	662.9 bA	656.4 aA	655.4 bA	670.4 bA	670.8 bA	672.6 bA	680.4 aA	677.0 bA
E21	698.2 aA	701.4 aA	703.9 aA	685.2 aA	710.3 aA	703.6 aA	704.8 aA	704.9 aA	701.3 aA	724.9 aA
E22	685.5 aA	672.6 bA	672.0 bA	666.3 aA	675.8 bA	668.4 bA	671.0 bA	671.4 bA	679.9 aA	677.3 bA
E23	676.9 aA	689.7 aA	681.0 bA	668.7 aA	678.0 bA	677.4 bA	681.6 bA	691.5 aA	685.6 aA	693.2 bA
E24	674.5 aA	687.9 aA	673.9 bA	683.7 aA	678.9 bA	679.7 bA	675.5 bA	677.9 bA	680.1 aA	684.1 bA
E25	680.7 aA	680.4 bA	673.7 bA	671.7 aA	672.9 bA	673.3 bA	672.3 bA	677.1 bA	680.7 aA	686.6 bA
E26	698.2 aA	701.4 aA	703.9 aA	685.2 aA	710.3 aA	703.6 aA	704.8 aA	704.9 aA	701.3 aA	724.9 aA
E27	687.1 aA	669.8 bA	667.7 bA	672.2 aA	668.8 bA	675.5 bA	679.1 bA	688.9 aA	686.1 aA	690.9 bA
E28	690.5 aA	688.8 aA	686.1 bA	673.7 aA	693.5 aA	686.9 bA	689.2 bA	667.7 bA	684.4 aA	693.1 bA
E29	675.4 aA	686.6 aA	672.4 bA	676.3 aA	675.0 bA	678.5 bA	674.6 bA	678.5 bA	675.6 aA	679.4 bA
E30	677.8 aA	673.7 bA	670.9 bA	667.7 aA	669.5 bA	672.1 bA	674.7 bA	674.3 bA	675.8 aA	684.2 bA

Environments (E) according to the schema proposed by Table 1. Cultivars: CD 2737 RR (1), BMX FLECHA 6266 RSF (2), NS 7209 IPRO (3), BMX FOCO 74I77 RSF IPRO (4), DM 75I76 RSF IPRO (5), ST 797 IPRO (6), BMX CHALLENGE 8473 RSF (7), BMX BONUS 8579 RSF IPRO (8), M7110 IPRO (9) and M-SOY 8866 (10). The averages followed by the same lower case letters in the column and upper case letters in the row did not differ statistically at 5% probability by the Scott Knott test. The averages followed by the different lowercase letters in the column and uppercase letters in the row differed statistically at 5% probability by the Scott Knott test.

### 3.4 Germination of stored soybean seeds

As is shown in Table 7, the germination percentages of seeds stored in uncoated packaging were reduced, mainly at ambient temperature storage conditions. Similar results were obtained for seeds stored at 10°C and 15°C temperatures, for up to six months. After the six-month period, the seeds stored at 10°C, both in coated and uncoated packaging, had better germination quality than the seeds stored at 15°C. The worst seed germination results were obtained after nine months of storage (Fig 2C). Coated packaging was efficient in preserving the quality of the stored seeds, as it reduced the effects of temperature and humidity variations. Among the cultivars, NS 7209 IPRO had the lowest germination percentage over the storage period.

**Table 7:** Breakdown of the interaction between storage environments and soybean cultivars for germination (%).

E/Cultivars	1	2	3	4	5	6	7	8	9	10
E1	100 aA	99 aA	64 cC	90 bB	94 aB	99 aA	99 aA	92 bB	99 aA	96 aA
E2	99 aA	96 aA	80 aB	94 aA	94 aA	100 aA	95 aA	98 aA	97 aA	97 aA
E3	93 aA	91 aA	41 fC	86 bB	81 bB	93 bA	98 aA	85 cB	96 aA	92 bA
E4	97 aA	86 bB	38 fE	86 bB	76 bC	99 aA	98 aA	78 dC	66 bD	75 dC
E5	69 bB	34 dD	20 hE	66 dB	50 dC	92 bA	92 aA	67 eB	17 cE	49 fC
E6	100 aA	99 aA	64 cC	90 bB	94 aB	99 aA	99 aA	92 bB	99 aA	96 aA
E7	99 aA	100 aA	61 cC	90 bB	87 aB	99 aA	96 aA	99 aA	97 aA	93 aB
E8	100 aA	99 aA	75 bD	98 aA	98 aA	87 bC	100 aA	93 bB	98 aA	93 aB
E9	99 aA	98 aA	57 dC	93 aB	93 aB	98 aA	100 aA	96 aA	99 aA	91 bB
E10	99 aA	96 aA	48 eD	95 aA	89 aB	100 aA	98 aA	91 bB	97 aA	82 cC
E11	100 aA	99 aA	64 cC	90 bB	94 aB	99 aA	99 aA	92 bB	99 aA	96 aA
E12	97 aA	99 aA	83 aB	92 bA	91 aA	95 bA	97 aA	96 aA	97 aA	94 aA
E13	99 aA	100 aA	78 bB	96 aA	99 aA	99 aA	100 aA	98 aA	99 aA	97 aA
E14	99 aA	97 aA	56 dB	97 aA	97 aA	100 aA	100 aA	98 aA	99 aA	96 aA
E15	94 aB	96 aA	73 bC	88 bB	93 aB	99 aA	98 aA	98 aA	100 aA	96 aA
E16	100 aA	99 aA	64 cC	90 bB	94 aB	99 aA	99 aA	92 bB	99 aA	96 aA
E17	99 aA	99 aA	60 cB	97 aA	96 aA	100 aA	98 aA	98 aA	95 aA	98 aA
E18	99 aA	96 aB	69 cC	93 aB	92 aB	99 aA	99 aA	99 aA	95 aB	99 aA
E19	93 aA	75 cC	29 gG	49 eF	69 cD	93 bA	97 aA	83 cB	61 bE	95 aA
E20	69 bB	7 eE	0 if	10 fE	13 eE	37 cD	77 bA	4 fF	1 dF	62 eC
E21	100 aA	99 aA	64 cC	90 bB	94 aB	99 aA	99 aA	92 bB	99 aA	96 aA
E22	98 aA	97 aA	81 aC	90 bB	93 aB	100 aA	94 aB	99 aA	99 aA	95 aB
E23	97 aA	98 aA	62 cC	95 aA	89 aB	100 aA	99 aA	95 aA	95 aA	96 aA
E24	98 aA	95 aA	60 cB	97 aA	93 aA	99 aA	99 aA	94 bA	95 aA	91 bA
E25	97 aA	95 aA	53 dC	88 bB	92 aB	98 aA	99 aA	92 bB	90 aB	87 cB
E26	100 aA	99 aA	64 cC	90 bB	94 aB	99 aA	99 aA	92 bB	99 aA	96 aA
E27	100 aA	100 aA	87 aB	79 cC	93 aA	100 aA	97 aA	97 aA	94 aA	96 aA
E28	100 aA	99 aA	73 bC	99 aA	97 aA	99 aA	100 aA	99 aA	97 aA	99 aA
E29	99 aA	96 aA	62 cB	99 aA	95 aA	99 aA	99 aA	94 bA	99 aA	97 aA
E30	98 aA	98 aA	75 bB	95 aA	93 aA	99 aA	100 aA	96 aA	98 aA	98 aA

Environments (E) according to the schema proposed by Table 1. Cultivars: CD 2737 RR (1), BMX FLECHA 6266 RSF (2), NS 7209 IPRO (3), BMX FOCO 74I77 RSF IPRO (4), DM 75I76 RSF IPRO (5), ST 797 IPRO (6), BMX CHALLENGE 8473 RSF (7), BMX BONUS 8579 RSF IPRO (8), M7110 IPRO (9) and M-SOY 8866 (10). The averages followed by the same lower case letters in the column and upper case letters in the row did not differ statistically at 5% probability by the Scott Knott test. The averages followed by the different lowercase letters in the column and uppercase letters in the row differed statistically at 5% probability by the Scott Knott test.

### 3.5 Electrical conductivity test on stored soybean seeds

The electrical conductivity results are shown in Table 8. Higher electrical conductivity values and, consequently, greater deterioration were obtained in soybean seeds stored in ambient temperature environments, when compared to the 10°C and 15°C environments (Table 7). Refrigerated environments reduced the deterioration of soybean seeds during the storage period (Fig 2D). Soybean seeds stored in uncoated packaging had the highest electrical conductivity values compared to seeds belonging to the same cultivar and stored under the same temperature in coated packaging and duration, thereby demonstrating the benefit of the latter packaging type in preserving seed quality. The 10°C environment helped preserve soybean seed quality better, and resulted in a solute leaching increase only after six months of storage, with emphasis on the NS 7209 IPRO and M-SOY 8866 cultivars.

**Table 8:** Breakdown of the interaction between storage environments and soybean cultivars for electrical conductivity (μS cm^-1^ of sample).

E/Cultivars	1	2	3	4	5	6	7	8	9	10
E1	82.5 dC	81.2 eC	145.5 gA	79.0 eC	90.6 fC	87.3 dC	73.2 cC	83.6 fC	89.1 fC	107.7 dB
E2	76.9 dC	84.3 eC	153.6 gA	83.5 eC	89.6 fC	76.2 dC	75.9 cC	112.5 dB	120.8 eB	114.6 cB
E3	84.8 dD	98.1 dC	189.3 eA	96.1 eC	105.3 eC	91.6 dD	80.9 cD	104.8 dC	128.5 dB	104.8 dC
E4	97.4 cD	131.2 cC	238.5 cA	127.2 cC	152.2 cB	106.8 cD	98.5 bD	131.2 cC	161.7 cB	125.4 cC
E5	116.4 bF	184.2 bC	281.4 bA	182.4 bC	191.9 bC	134.2 bE	111.6 bF	172.7 bC	207.9 bB	156.8 bD
E6	82.5 dC	81.2 eC	145.5 gA	79.0 eC	90.6 fC	87.3 dC	73.2 cC	83.6 fC	89.1 fC	107.7 dB
E7	72.0 dC	74.7 eC	136.9 hA	77.6 eC	79.6 fC	76.8 dC	65.5 cC	81.5 fC	92.3 fB	95.4 dB
E8	87.9 dC	99.6 dC	167.3 fA	92.4 eC	108.6 eB	93.7 dC	83.9 cC	93.4 eC	115.6 eB	121.6 cB
E9	81.4 dC	99.3 dC	164.1 fA	99.8 eC	105.7 eC	86.9 dD	80.9 cD	97.8 eC	116.4 eB	122.4 cB
E10	80.6 dC	100.5 dD	190.2 eA	110.2 dC	109.1 eC	94.7 dD	87.1 cD	106.6 dC	129.9 dB	117.1 cC
E11	82.5 dC	81.2 eC	145.5 gA	79.0 eC	90.6 fC	87.3 dC	73.2 cC	83.6 fC	89.1 fC	107.7 dB
E12	63.8 dC	71.2 eC	132.5 hA	76.8 eC	89.4 fB	69.4 dC	72.7 cC	74.7 fC	94.8 fB	88.9 dB
E13	86.8 dC	88.3 eC	167.7 fA	90.8 eC	100.6 eB	86.3 dC	79.1 cC	94.8 eC	108.4 eB	106.7 dB
E14	76.8 dC	87.2 eC	160.0 fA	85.4 eC	103.3 eB	82.9 dC	76.5 cC	93.9 eC	106.5 eB	112.1 dB
E15	79.6 dC	89.7 eC	166.9 fA	86.4 eC	101.3 eB	87.5 dC	80.3 cC	88.7 eC	101.8 fB	107.1 dB
E16	82.5 dC	81.2 eC	145.5 gA	79.0 eC	90.6 fC	87.3 dC	73.2 cC	83.6 fC	89.1 fC	107.7 dB
E17	81.5 dC	91.1 eC	182.1 eA	92.9 eC	102.7 eB	77.3 dC	82.1 cC	103.9 dB	118.3 eB	107.7 dB
E18	78.2 dC	79.9 eC	178.3 eA	82.4 eC	105.9 eB	82.3 dC	75.5 cC	93.1 eC	114.3 eB	102.1 db
E19	97.1 cD	105.2 dD	239.7 cA	123.2 cC	139.1 dC	119.0 bC	95.5 bD	130.5 cC	193.4 bB	125.3 cC
E20	193.0 aD	258.6 aB	313.5 aA	220.7 aC	246.3 aB	223.4 aC	160.2 aE	214.0 aC	303.5 aA	189.5 aD
E21	82.5 dC	81.2 eC	145.5 gA	79.0 eC	90.6 fC	87.3 dC	73.2 cC	83.6 fC	89.1 fC	107.7 dB
E22	72.3 dC	78.8 eC	148.6 gA	77.7 eC	81.6 fC	82.2 dC	68.6 cC	73.4 fC	95.6 fB	104.3 dB
E23	74.2 dC	88.9 eC	157.3 gA	89.6 eC	104.9 eB	85.7 dC	82.6 cC	90.8 eC	100.5 fB	112.0 dB
E24	88.6 dE	93.5 dE	190.8 eA	105.1 dD	113.2 eD	94.1 dE	87.6 cE	106.5 dD	155.6 cB	125.6 cC
E25	100.5 cD	105.1 dD	215.9 dA	126.3 cC	131.2 dC	102.9 cD	99.5 bD	123.9 cC	151.6 cB	150.1 bB
E26	82.5 dC	81.2 eC	145.5 gA	79.0 eC	90.6 fC	87.3 dC	73.2 cC	83.6 fC	89.1 fC	107.7 dB
E27	69.4 dD	72.0 eD	126.6 hA	78.0 eC	83.9 fC	70.1 dD	66.7 cD	72.3 fD	88.5 fC	97.9 dB
E28	72.9 dC	79.8 eC	155.0 gA	82.7 eC	106.9 eB	82.9 dC	80.9 cC	79.6 fC	109.9 eB	117.6 cB
E29	75.8 dC	79.0 eC	164.4 fA	85.8 eC	86.8 fC	80.1 dC	75.8 cC	88.5 eC	118.6 eB	109.2 dB
E30	74.6 dC	81.0 eC	163.6 fA	87.9 eC	104.0 eB	89.9 dC	81.5 cC	91.5 eC	94.5 fC	115.2 cB

Environments (E) according to the schema proposed by Table 1. Cultivars: CD 2737 RR (1), BMX FLECHA 6266 RSF (2), NS 7209 IPRO (3), BMX FOCO 74I77 RSF IPRO (4), DM 75I76 RSF IPRO (5), ST 797 IPRO (6), BMX CHALLENGE 8473 RSF (7), BMX BONUS 8579 RSF IPRO (8), M7110 IPRO (9) and M-SOY 8866 (10). The averages followed by the same lower case letters in the column and upper case letters in the row did not differ statistically at 5% probability by the Scott Knott test. The averages followed by the different lowercase letters in the column and uppercase letters in the row differed statistically at 5% probability by the Scott Knott test.

### 3.6 First germination count (vigor) of stored soybean seeds

The first germination count of soybean seeds was better in the 15°C and 10°C storage environments than in the ambient temperature environments. These results were similar to those obtained from the electrical conductivity and germination assessments, which were favorable in the 15°C and 10°C storage environments and with the use of coated packaging (Table 9). Over the storage period, we observed reductions in the percentage of the first count of germinated seeds, with greater emphasis on storage at ambient temperature (Fig 2E).

**Table 9:** Breakdown of the interaction between storage environments and soybean cultivars for the 1st germination count (%).

E/Cultivars	1	2	3	4	5	6	7	8	9	10
E1	99 aA	95 aA	50 cC	84 bB	87 aB	94 aA	97 aA	84 bB	93 aA	95 aA
E2	97 aA	89 bB	76 aC	93 aB	83 aC	98 aA	88 bB	97aA	90 aB	95 aA
E3	72 cB	56 dC	18 fE	45 fD	49 dD	67 cB	88 bA	43 eD	48 dD	66 cB
E4	97 aA	85 bB	38 dE	86 bB	56 cC	99 aA	97 aA	47 eD	53 dC	56 dC
E5	47 dB	14 eD	11 fD	23 gC	18 eC	50 dB	58 dA	12 fD	10 fD	26 fC
E6	99 aA	95 aA	50 cC	84 bB	87 aB	94 aA	97 aA	84 bB	93 aA	95 aA
E7	96 aA	98 aA	48 cF	63 dE	62 cE	99 aA	72 cD	88 aB	82 bC	77 bC
E8	95 aA	69 cC	44 cD	78 cB	88 aA	73 cC	94 aA	69 cC	79 bB	77 bB
E9	98 aA	97 aA	50 cC	86 bB	82 aB	97 aA	97 aA	91 aB	86 aB	85 bB
E10	95 aA	83 bB	39 dE	73 cC	67 bD	90 aA	82 bB	78 cC	86 aB	66 cD
E11	99 aA	95 aA	50 cC	84 bB	87 aB	94 aA	97 aA	84 bB	93 aA	95 aA
E12	89 bA	97 aA	73 aC	86 bB	51 dD	85 bB	93 aA	83 bB	88 aA	94 aA
E13	92 aA	88 bA	56 cC	80 bB	84 aB	84 bB	89 bA	79 bB	76 bB	83 bB
E14	99 aA	94 aB	48 cC	91 aB	90 aB	99 aA	93 aB	91 aB	90 aB	89 aB
E15	87 bB	88 bB	58 cD	69 dC	82 aB	97 aA	86 bB	92 aA	85 aB	91 aA
E16	99 aA	95 aA	50 cC	84 bB	87 aB	94 aA	97 aA	84 bB	93 aA	95 aA
E17	98 aA	95 aA	44 cC	96 aA	90 aA	96 aA	95 aA	92 aA	81 bB	93 aA
E18	79 cB	71 cB	38 dD	58 eC	61 cC	71 cB	86 bA	73 cB	57 cC	90 aA
E19	85 bA	63 dC	28 eF	40 fE	60 cC	83 bA	88 bA	74 cB	49 dD	87 bA
E20	30 eC	0 fD	0 gD	4 hD	4 fD	11 eD	60 dA	1 gD	0 gD	47 eB
E21	99 aA	95 aA	50 cC	84 bB	87 aB	94 aA	97 aA	84 bB	93 aA	95 aA
E22	97 aA	91 aB	75 aC	70 dC	86 aB	98 aA	90 bB	95 aA	93 aB	90 aB
E23	90 bA	74 cB	32 eE	50 eD	64 cC	85 bA	88 bA	50 eD	62 cC	78 bB
E24	93 aA	83 bB	49 cC	76 cB	82 aB	95 aA	95 aA	80 bB	85 aB	82 bB
E25	55 dC	57 dC	34 eD	54 eC	75 bB	85 bA	92 aA	56 dC	29 eD	59 cC
E26	99 aA	95 aA	50 cC	84 bB	87 aB	94 aA	97 aA	84 bB	93 aA	95 aA
E27	98 aA	100 aA	82 aB	64 dC	87 aB	97 aA	90 bB	93 aA	89 aB	96 aA
E28	90 bA	85 bA	49 cD	68 dC	80 aB	90 aA	88 bA	76 cB	74 bB	82 bB
E29	98 aA	95 aA	54 cB	95 aA	89 aA	97 aA	96 aA	90 aA	91 aA	94 aA
E30	89 bA	84 bB	63 bC	82 bB	88 aA	97 aA	94 aA	79 bB	90 aA	91 aA

Environments (E) according to the schema proposed by Table 1. Cultivars: CD 2737 RR (1), BMX FLECHA 6266 RSF (2), NS 7209 IPRO (3), BMX FOCO 74I77 RSF IPRO (4), DM 75I76 RSF IPRO (5), ST 797 IPRO (6), BMX CHALLENGE 8473 RSF (7), BMX BONUS 8579 RSF IPRO (8), M7110 IPRO (9) and M-SOY 8866 (10). The averages followed by the same lower case letters in the column and upper case letters in the row did not differ statistically at 5% probability by the Scott Knott test. The averages followed by the different lowercase letters in the column and uppercase letters in the row differed statistically at 5% probability by the Scott Knott test.

### 3.7 Mechanical damage from moisture in stored soybean seeds

The tetrazolium test is based on the activity of the dehydrogenase enzymes, particularly malic acid dehydrogenase, which reduces the 2,3,5 triphenyl tetrazolium chloride salt in the living tissue of the seed, where hydrogen ions are transferred to the said salt. When the seed is immersed in the tetrazolium solution, it diffuses through the tissues, occurring in the living cells, the reduction reaction, resulting in the formation of a red, non-diffusible compound, known as triphenylformazan, indicating that there is respiratory activity in the mitochondria and, consequently, that the tissue is viable (alive). Dead tissues (not viable) do not react with the solution preserving its natural color.

Moisture damage caused by the tetrazolium test was related to the death of soybean seeds and the consequent loss of viability, which were greater at nine months of storage at ambient temperature compared to the other storage duration and packaging conditions applied to the same cultivar. During the storage period, the moisture damage percentage of the soybean cultivars increased (Table 10). Storage at ambient temperature and in uncoated packaging resulted in the greatest moisture and heat exchange between the seed mass and the intergranular storage air over time, thereby intensifying seed deterioration.

**Table 10:** Breakdown of the interaction between storage environments and soybean cultivars on moisture damage (%) obtained by analyzing the tetrazolium test.

E/Cultivars	1	2	3	4	5	6	7	8	9	10
E1	3 cA	3 eA	5 gH	0 hB	2 dB	1 eB	2 cB	1 eB	2 eB	0 dB
E2	2 cA	0 fA	0 hA	0 hA	0 eA	0 eA	0 dA	1 eA	0 fA	0 dA
E3	0 dA	2 eA	1 hA	0 hA	0 eA	0 eA	2 cA	0 eA	0 fA	0 dA
E4	12 bB	15 cA	7 fC	0 hD	0 eD	0 eD	0 dD	2 eD	7 cC	1 dD
E5	10 bG	35 bC	56 bB	65 aA	35 bC	18 bE	15 bF	54 bB	34 bC	24 aD
E6	3 cA	3 eA	5 gA	0 hB	2 dB	1 eB	2 cB	1 eB	2 eB	0 dB
E7	0 dA	0 fA	1 hA	1 hA	2 dA	1 eA	0 dA	1 eA	0 fA	1 dA
E8	0 dB	1 fB	4 gA	1 hB	1 eB	2 eA	0 dB	2 eA	0 fB	1 dB
E9	0 dB	0 fB	5 gA	0 hB	2 dB	0 eB	0 dB	1 eB	0 fB	1 dB
E10	3 cD	6 dC	9 eB	14 dA	0 eE	6 cC	2 cD	12 cA	4 dC	4 cC
E11	3 cA	3 eA	5 gA	0 hB	2 dB	1 eB	2 cB	1 eB	2 eB	0 dB
E12	0 dB	0 fB	4 gA	1 hB	1 eB	0 eB	0 dB	0 eB	0 fB	0 dB
E13	1 dB	0 fB	1 hB	3 gA	1 eB	0 eB	0 dB	3 eA	0 fB	5 cA
E14	0 dC	1 fC	14 dA	4 gB	1 eC	1 eC	1 cC	2 eC	0 fC	0 dC
E15	1 dE	0 fE	19 cA	8 eB	5 dC	3 dC	0 dE	2 eD	1 eE	3 cD
E16	3 cA	3 eA	5 gA	0 hB	2 dB	1 eB	2 cB	1 eB	2 eB	0 dB
E17	0 dB	0 fB	0 hB	5 fA	0 eB	0 eB	0 dB	5 dA	1 eB	0 dB
E18	0 dB	1 fB	5 gA	2 gB	0 eB	1 eB	0 dB	3 eA	2 eB	1 dB
E19	3 cB	3 eB	8 fA	0 hC	2 dB	0 eC	0 dC	1 eC	1 eC	2 cB
E20	30 aG	85 aA	87 aA	40 bE	76 aB	33 aF	49 aD	77 aB	72 aC	11 bH
E21	3 cA	3 eA	5 gA	0 hB	2 dB	1 eB	2 cB	1 eB	2 eB	0 dB
E22	0 dB	0 fB	5 gA	0 hB	2 db	0 eB	0 dB	0 eB	0 fB	0 dB
E23	0 dA	1 fA	4 gA	1 hA	1 eA	2 eA	1 cA	1 eA	2 eA	1 dA
E24	0 dB	2 eB	5 gA	1 hB	3 dA	0 eB	0 dB	1 eB	1 eB	1 dB
E25	0 dE	16 cB	11 eC	35 cA	10 cC	3 dD	1 cE	5 dD	0 fE	10 bC
E26	3 cA	3 eA	5 gA	0 hB	2 dB	1 eB	2 cB	1 eB	2 eB	0 dB
E27	0 dA	1 fA	0 hA	2 gA	0 eA	0 eA	0 dA	0 eA	0 fA	0 dA
E28	0 dA	0 fA	1 hB	0 hA	0 eA	0 eA	0 dA	1 eA	2 eA	2 cA
E29	0 dB	0 fB	2 hB	6 fA	2 dB	0 eB	0 dB	5 dA	0 fB	1 dB
E30	0 dE	2 eD	13 dA	4 gD	10 cB	0 eE	0 dE	7 dC	1 eE	3 cD

Environments (E) according to the schema proposed by Table 1. Cultivars: CD 2737 RR (1), BMX FLECHA 6266 RSF (2), NS 7209 IPRO (3), BMX FOCO 74I77 RSF IPRO (4), DM 75I76 RSF IPRO (5), ST 797 IPRO (6), BMX CHALLENGE 8473 RSF (7), BMX BONUS 8579 RSF IPRO (8), M7110 IPRO (9) and M-SOY 8866 (10). The averages followed by the same lower case letters in the column and upper case letters in the row did not differ statistically at 5% probability by the Scott Knott test. The averages followed by the different lowercase letters in the column and uppercase letters in the row differed statistically at 5% probability by the Scott Knott test.

The package coating contributed to the reduction of the moisture damage percentage of soybean seeds after nine months in ambient temperature and 15°C storage conditions, compared to the same cultivar, with the same storage duration, and the use of uncoated packaging. However, the best storage conditions were at 10°C, as these resulted in reduced moisture damage. The water content and temperature influenced the respiratory activity of the stored seeds and their effects on the deterioration process, an effect that became more evident with the storage duration increase (Fig 2F).

The desorption or sorption of water depending on the storage conditions caused a variation in the seeds water content reaching different humidity levels of hygroscopic balance between the seeds and the storage environment. This variation of the moisture led a disruption of cellular tissues causing physical damage and deterioration of the seeds. The damage caused by the variation of humidity interfered in the physiological quality of the seeds during the storage time. The tetrazolium test characterized the damage caused by moisture. The seed genotypes influenced the sorption and desorption of water content and, consequently, physical damage.

### 3.8 Tetrazolium vigor test on stored soybean seeds

The best results in terms of preserving vigor during storage were obtained at the 10°C storage environment; similar results were obtained at the 15°C storage environment (Table 11). The worst results were obtained under ambient temperature storage conditions, regardless of the use of package coating. Package coating helped slow down the deterioration progress and preserve seed vigor in the 15°C and 10°C storage environments, for the same cultivar and storage duration. The cold storage contributed to the preservation of the physiological quality of soybean seeds, with the difference between this and the ambient temperature storage being clearer after six months of storage (Fig 2G).

**Table 11:** Breakdown of the interaction between storage environments and soybean cultivars on the vigor test (%) obtained by analyzing the tetrazolium test.

E/Cultivars	1	2	3	4	5	6	7	8	9	10
E1	89 cA	83 cA	75 bB	63 fC	85 bA	81 cB	87 bA	58 eC	77 cB	85 cA
E2	91 cA	70 eC	29 gD	86 cB	70 cC	92 aA	83 cB	76 cC	73 dC	86 cB
E3	92 bA	90 bA	57 dD	86 cB	64 dC	90 bA	95 aA	81 bB	84 bB	87 cB
E4	68 fB	55 fD	29 gE	84 cA	61 dC	85 cA	82 cA	50 fD	66 eB	86 cA
E5	52 gC	20 gE	16 hE	34 gD	33 eD	71 eA	61 eB	5 gF	31 gD	35 hD
E6	89 cA	83 cA	75 bB	63 fC	85 bA	81 cB	87 bA	58 eC	77 cB	85 cA
E7	94 bA	91 bA	62 cC	87 cA	92 aA	92 aA	90 bA	61 dC	69 dB	95 aA
E8	86 cB	95 aA	55 dD	86 cB	93 aA	94 aA	93 aA	91 aA	64 eC	90 bA
E9	86 cA	86 cA	56 dD	92 bA	86 bA	84 cA	89 bA	64 dC	74 dB	86 cA
E10	91 cB	80 cC	74 bC	72 eD	96 aA	75 dC	78 dC	66 dE	79 cC	89 bB
E11	89 cA	83 cA	75 bB	63 fC	85 bA	81 cB	87 bA	58 eC	77 cB	85 cA
E12	83 dA	76 dB	52 dD	90 bA	68 cC	87 bA	73 dB	54 fD	64 eC	88 cA
E13	98 aA	83 cC	74 bD	97 aA	86 bC	93 aB	99 aA	96 aA	84 bC	91 bB
E14	82 dC	98 aA	59 dE	74 eD	85 bC	90 bB	84 cC	74 cD	82 bC	77 dD
E15	98 aA	88 cB	58 dD	79 dC	86 bB	88 bB	82 cC	91 aA	86 bB	94 aA
E16	89 cA	83 cA	75 bB	63 fC	85 bA	81 cB	87 bA	58 eC	77 cB	85 cA
E17	93 bA	87 cB	44 fF	78 dC	73 cD	88 bB	83 cC	54 fE	74 dD	97 aA
E18	86 cC	85 cC	42 fE	98 aA	74 cD	84 cC	75 dD	82 bC	90 aB	96 aA
E19	74 eB	68 eC	43 fE	84 cA	75 cB	76 dB	76 dB	60 eD	42 fE	72 eB
E20	28 hB	0 hD	1 iD	13 hC	1 fD	13 fC	13 fC	0 gD	0 hD	53 gA
E21	89 cA	83 cA	75 bB	63 fC	85 bA	81 cB	87 bA	58 eC	77 cB	85 cA
E22	77 dC	69 eD	76 bC	81 dB	81 bB	88 bA	88 bA	82 bB	74 cC	87 cA
E23	92 bA	88 cA	87 aA	91 bA	89 aA	96 aA	79 dB	73 cC	62 eD	89 bA
E24	80 dB	81 cB	49 eE	91 bA	74 cC	83 cB	80 dB	67 dD	76 cC	80 dB
E25	93 bA	74 dC	64 cD	63 fD	70 cC	84 cB	86 bB	75 cC	80 cB	67 fD
E26	89 cA	83 cA	75 bB	63 fC	85 bA	81 cB	87 bA	58 eC	77 cB	85 cA
E27	85 cB	67 eC	40 fE	62 fD	71 cC	66 eC	92 aA	62 dD	65 eD	94 aA
E28	98 aA	97 aA	75 bC	97 aA	92 aB	94 aB	96 aA	80 bC	90 aB	93 aB
E29	87 cA	87 cA	58 dD	76 eB	80 bB	81 cB	83 cB	71 cC	73 dC	81 dB
E30	92 bA	85 cB	59 dC	86 cB	63 dC	89 bA	88 bA	80 bB	83 bB	91 bA

Environments (E) according to the schema proposed by Table 1. Cultivars: CD 2737 RR (1), BMX FLECHA 6266 RSF (2), NS 7209 IPRO (3), BMX FOCO 74I77 RSF IPRO (4), DM 75I76 RSF IPRO (5), ST 797 IPRO (6), BMX CHALLENGE 8473 RSF (7), BMX BONUS 8579 RSF IPRO (8), M7110 IPRO (9) and M-SOY 8866 (10). The averages followed by the same lower case letters in the column and upper case letters in the row did not differ statistically at 5% probability by the Scott Knott test. The averages followed by the different lowercase letters in the column and uppercase letters in the row differed statistically at 5% probability by the Scott Knott test.

### 3.9 Viability test with tetrazolium in stored soybean seeds

The tetrazolium test performed to evaluate the viability percentage of the seeds produced similar results in all treatments, over the storage period (Table 12). The best results regarding the viability of the seeds were obtained from the 10°C and 15°C seed storage conditions up to twelve months (Fig 2H). The package coating contributed to the preservation of the viability percentage of the seeds.

**Table 12:** Breakdown of the interaction between storage environments and soybean cultivars on the viability test (%) obtained by analyzing the tetrazolium test.

E/Cultivars	1	2	3	4	5	6	7	8	9	10
E1	93 bA	95 bA	85 cB	91 bA	94 bA	93 bA	93 bA	91 cA	88 cB	91 bA
E2	95 bB	93 bB	87 cC	98 aA	96 aB	100 aA	95 bB	90 cC	83 dD	94 aB
E3	97 aB	94 bB	94 aB	99 aA	96 aB	100 aA	95 bB	99 aA	86 cC	96 aB
E4	85 cB	85 dB	87 cB	97 aA	95 bA	99 aA	94 bA	98 aA	82 dB	96 aA
E5	84 cA	64 fC	40 fF	34 fG	62 eC	81 cA	75 eB	46 dE	63 eC	58 cD
E6	93 bA	95 bA	85 cB	91 bA	94 bA	93 bA	93 bA	91 cA	88 cB	91 bA
E7	99 aA	94 bA	85 cB	96 aA	93 bA	96 bA	95 bA	95 bA	81 dC	95 aA
E8	96 bA	99 aA	79 dC	97 aA	97 aA	97 aA	96 bA	98 aA	86 cB	100 aA
E9	98 aA	99 aA	89 bC	99 aA	95 bB	98 aA	96 bB	94 bB	89 cC	95 aB
E10	97 aA	89 cC	88 bC	81 cE	100 aA	91 bC	85 dD	88 cC	87 cC	94 aB
E11	93 bA	95 bA	85 cB	91 bA	94 bA	93 bA	93 bA	91 cA	88 cB	91 bA
E12	98 aA	97 aA	90 bB	97 aA	95 bA	95 bA	94 bA	87 cB	94 bA	95 aA
E13	99 aA	97 aA	94 aB	97 aA	96 aA	100 aA	99 aA	97 aA	91 bB	91 bB
E14	98 aA	100 aA	86 cC	96 aA	95 bA	98 aA	93 bB	97 aA	92 bB	96 aA
E15	99 aA	100 aA	76 eD	89 bC	94 bB	95 bB	91 cC	98 aA	89 cC	96 aB
E16	93 bA	95 bA	85 cB	91 bA	94 bA	93 bA	93 bA	91 cA	88 cB	91 bA
E17	96 bA	96 bA	82 dD	94 aA	96 aA	95 bA	91 cB	90 cB	86 cC	99 aA
E18	98 aA	96 bA	88 bC	98 aA	97 aA	99 aA	91 cB	94 bB	93 bB	96 aA
E19	96 bA	92 bB	87 cC	95 aB	96 aA	99 aA	86 dC	98 aA	93 bB	94 aB
E20	66 dB	15 gG	13 gG	58 eC	24 fF	64 dB	50 fD	23 eF	28 fE	86 bA
E21	93 bA	95 bA	85 cB	91 bA	94 bA	93 bA	93 bA	91 cA	88 cB	91 bA
E22	96 bA	95 bA	86 cB	96 aA	92 bA	93 bA	100 aA	96 bA	83 dB	96 aA
E23	99 aA	97 aA	94 aA	97 aA	95 bA	98 aA	89 cB	97 aA	90 bB	95 aA
E24	96 bA	94 bA	86 cB	96 aA	94 bA	99 aA	93 bA	96 bA	88 cB	96 aA
E25	99 aA	79 eD	88 bC	64 dE	87 cC	95 bB	91 cC	95 bB	98 aA	89 bC
E26	93 bA	95 bA	85 cB	91 bA	94 bA	93 bA	93 bA	91 cA	88 cB	91 bA
E27	94 bB	93 bB	88 bC	94 aB	95 bA	98 aA	96 bA	99 aA	92 bB	97 aA
E28	99 aA	98 aA	91 bB	98 aA	99 aA	100 aA	99 aA	97 aA	94 bB	97 aA
E29	99 aA	99 aA	91 bB	93 bB	93 bB	99 aA	93 bB	94 bB	90 bB	96 aA
E30	100 aA	95 bB	81 dD	95 aB	80 dD	97 aA	94 bB	93 cB	89 cC	95 aB

Environments (E) according to the schema proposed by Table 1. Cultivars: CD 2737 RR (1), BMX FLECHA 6266 RSF (2), NS 7209 IPRO (3), BMX FOCO 74I77 RSF IPRO (4), DM 75I76 RSF IPRO (5), ST 797 IPRO (6), BMX CHALLENGE 8473 RSF (7), BMX BONUS 8579 RSF IPRO (8), M7110 IPRO (9) and M-SOY 8866 (10). The averages followed by the same lower case letters in the column and upper case letters in the row did not differ statistically at 5% probability by the Scott Knott test. The averages followed by the different lowercase letters in the column and uppercase letters in the row differed statistically at 5% probability by the Scott Knott test.

### 3.10 Cluster and principal component analysis of the environments, storage time, and soybean cultivars

**Fig 3:**
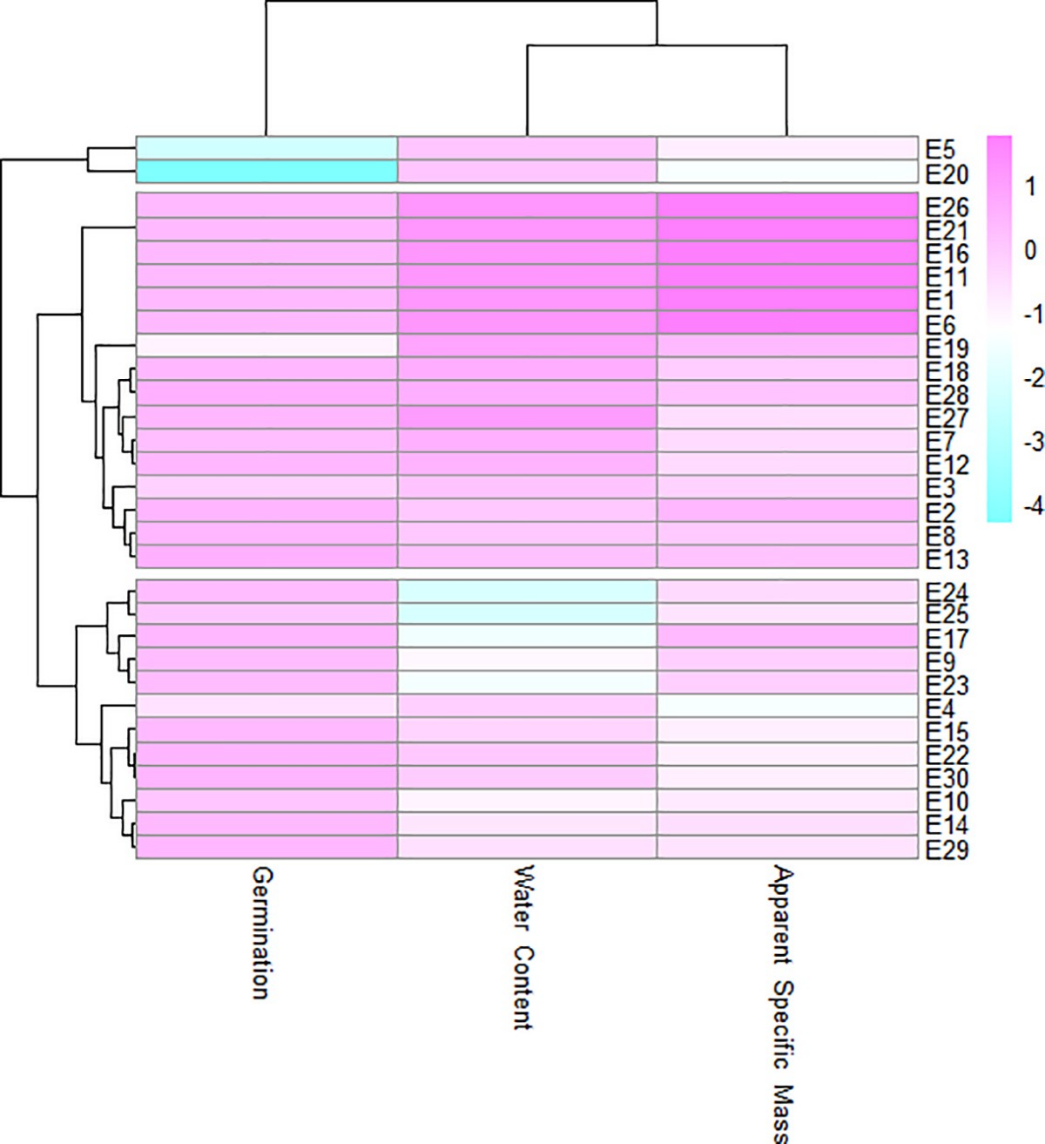
Cluster analysis of storage environments on the effects of physical and physiological quality of soybean seeds.

**Fig 4:**
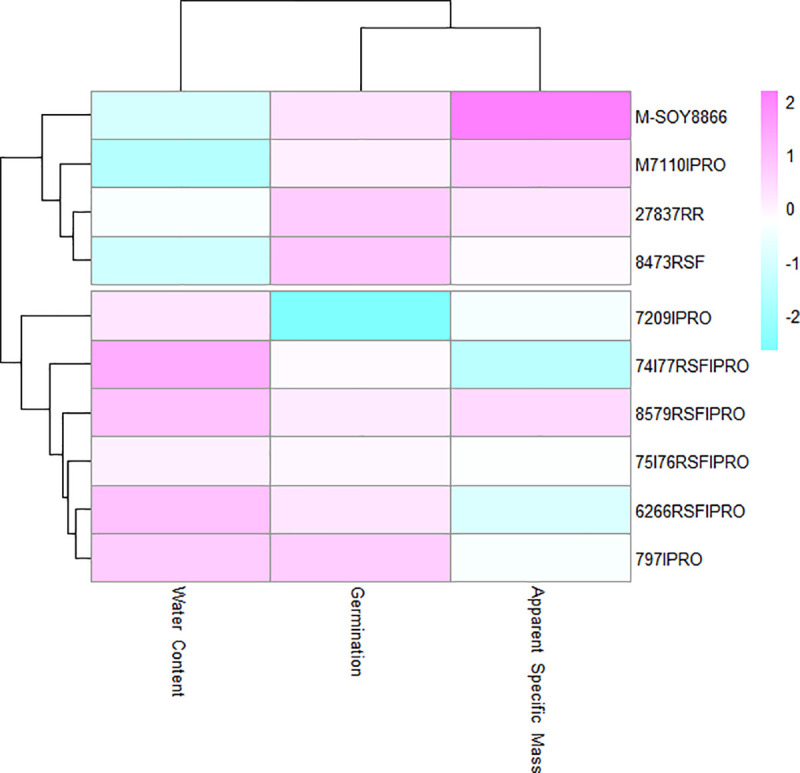
Cluster analysis of soybean cultivars on the effects of physical and physiological seed quality.

In the cluster analysis, values close to red and blue indicated higher and lower means, respectively, for the germination, water content, and specific mass in the environment (Fig 3) or in the cultivar variables (Fig 4).

**Fig 5:**
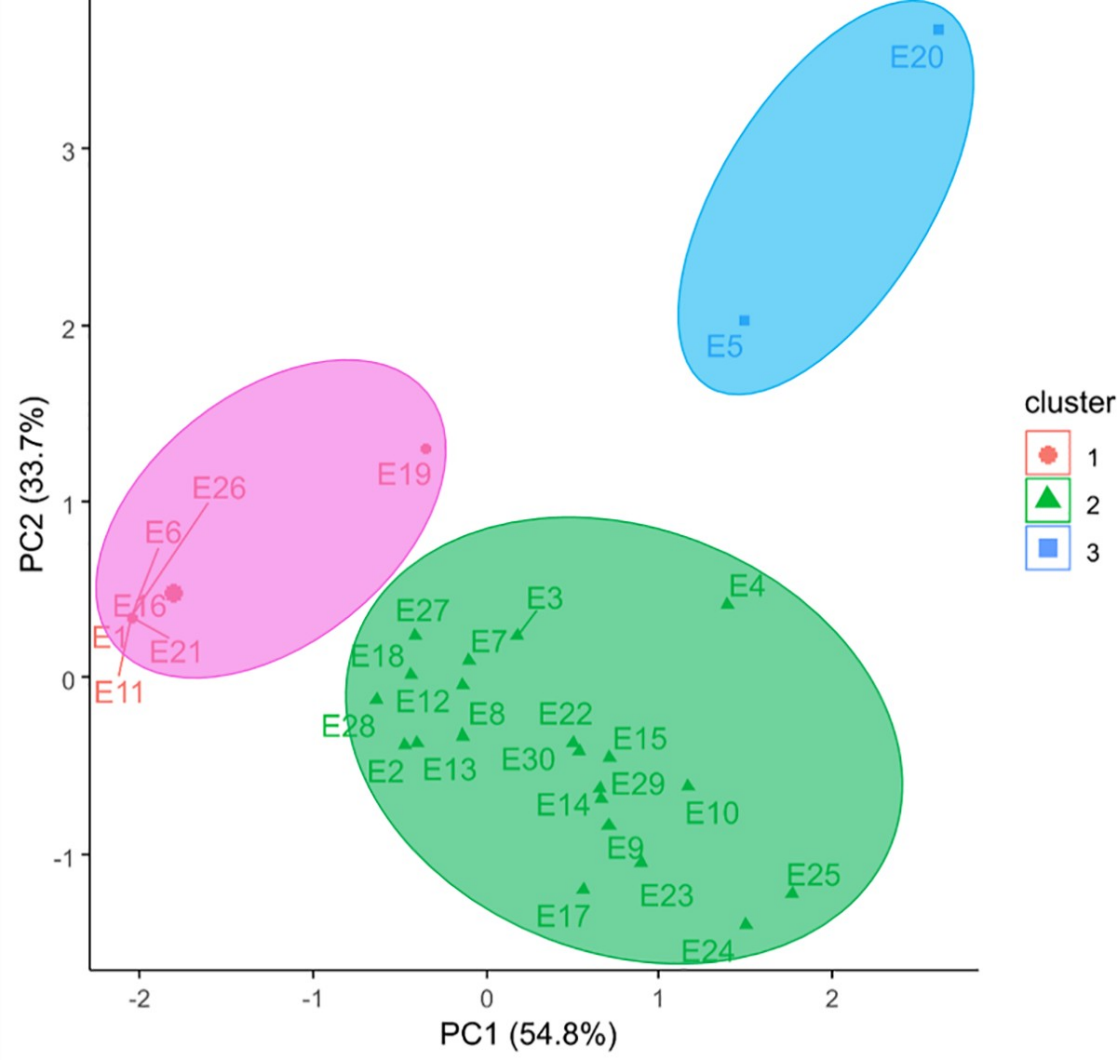
Principal component analysis of soybean seed storage environments.

As is shown in Fig 3, the group formed by the E5 and E20 environments had the lowest germination values and intermediate water content and specific mass values. These results were confirmed by the principal component analysis (Fig 5), in which the environments were arranged in cluster 3.

Greater deterioration was observed in seeds stored at ambient temperature than in cooled environments, with all the other variables remaining the same (same cultivar, package coating, and storage duration). The E5 (ambient temperature, with the use of package coating, and twelve months of storage) and E20 (ambient temperature, without the use of package coating, and twelve months of storage) environments stood out as the worst in terms of the physiological quality results obtained (Fig 3).

**Fig 6:**
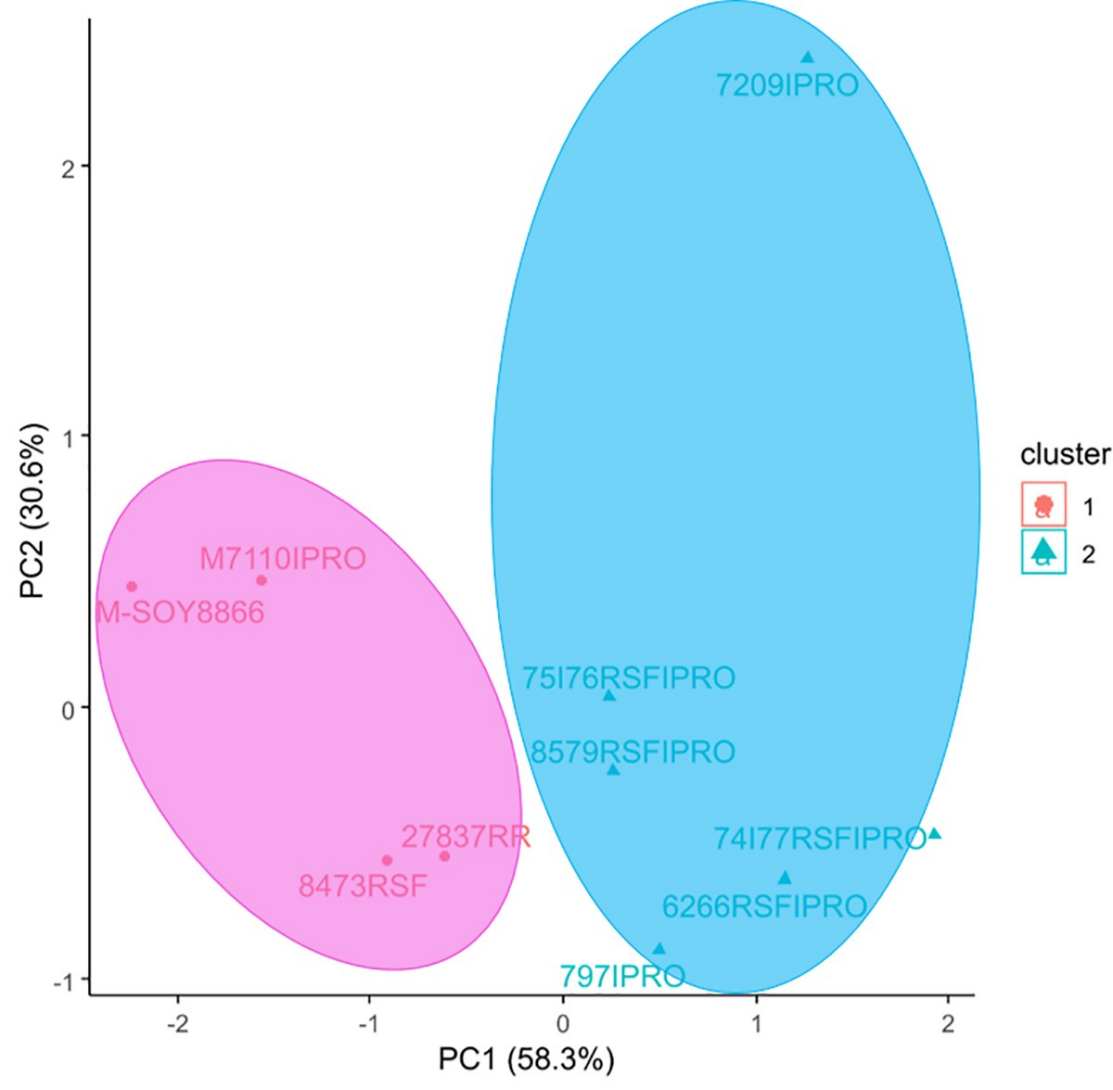
Principal component analysis of soybean cultivars in storage environments.

The use of coated packaging was beneficial in preserving the physiological quality of stored soybean seeds, however, its effect was significant only in ambient temperature environments for the same cultivar, type of package coating, and storage duration (Fig 3). Among the storage environments allocated in group 2 (Fig 3), E1, E6, E11, E16, E21, and E26 stood out for resulting in the highest germination, water content, and specific mass averages. These environments were also allocated to group 2 of the principal component analysis (Fig 6), thereby confirming their similarity in terms of resulting in the highest averages for soybean cultivars for the initial storage conditions (time point 0).

There was agreement regarding the formation of groups in the cluster (Fig 4) and main component (Fig 6) analyses applied to soybean cultivars. The M-SOY8866, M7110IPRO, CD 2737 RR, and BMX DESAFIO 8473 RSF cultivars were allocated to the group and stood out for resulting in the highest apparent specific mass and germination values as well as in the lower water content values. The other cultivars were allocated to group 2. In the storage environment grouping in the assessment of the physiological quality of soybean seed cultivars, the best results were obtained from environments E1, E6, E11, E16, E21, and E26 that represented time point 0 of storage (Fig 5).

The worst storage environment results were obtained from E5 and E20, which represented twelve months of storage in ambient temperature with and without the use of coating, respectively, thereby indicating that the longest storage duration and the ambient temperature were the factors that affected the physiological quality of soybean seeds in the most negative manner (Fig 5). The performance of stored soybean cultivars may differ under some conditions owing to genetic diversity; however, storage conditions can preserve the physiological quality of seeds (Fig 5). The thermal stability of seeds in the 10°C and 15°C storage environments was similar (Fig 5).

Different results were obtained depending on the storage conditions of soybean seed lots. However, depending on the cultivar, this difference was more evident in terms of the storage duration. The cultivars were subjected to different temperature and packaging conditions during twelve months of storage and, despite responding similarly to unfavorable conditions, owing to their specific characteristics, the soybean seeds of the M-SOY 8866, M7110 IPRO, CD 2737 RR, and BMX CHALLENGE 8473 RSF cultivars had the best performances in the physiological quality tests, which were performed in different storage environments (Fig 6).

The identification of the behavioral patterns of different cultivars in different storage environments facilitated our understanding of which of these environments were the most appropriate for storage (Fig 6). Our results allowed us to observe that the genetic characteristics of the cultivars and the environmental effects during the different storage stages, influenced seed viability (Fig 6).

## 4. Discussion

### 4.1 Water content of stored soybean seeds

In a study by Hartmann Filho et al. [18], the storage of soybeans in an uncontrolled environment resulted in an increase in their water content at 45 days and six months; however, a water content reduction was observed between three and five months [8]. Smaniotto et al. [19] observed a reduction in the water content of soybean seeds stored for six months at an average temperature of 27°C. Zuffo et al. [20] found a reduction in the water content of soybean seeds stored for eight months in a non-conditioned environment.

According to Conceição et al. [21], the water content of soybean seeds stored in a non-conditioned environment decreases from 11.1% (w.b.) to 10.0% (w.b.). A similar behavior has been observed in crambe seeds stored for nine months, during which period it was possible to contain water within the metallic packaging, while this was not possible with braided polypropylene, polyethylene terephthalate (PET), bottle, and styrofoam box packaging [22]. Another study showed that within three months of storage in an air-conditioned environment, big bag packaging allowed greater water and temperature conservation in soybean seeds, compared to Kraft paper packaging [23].

The storage of soybean seeds with a water content of 11% allowed for better preservation of their physiological quality, however the best results were obtained at lower temperatures. According to Alencar et al. [24], the association of higher temperatures and water content may increase the deterioration rate of soybeans. Zuffo et al. [20] found a reduction in the water content of soybean seeds stored for eight months in a non-climatized environment. In the work carried out by Juvino et al. [25], the soybean seeds that were stored for nine months showed greater amplitude in terms of their water content variation in the non-climatized environment than in the ambient environment (18°C) owing to the greater influence of the temperature and relative humidity changes. Zucarelli et al. [26] found higher water contents in bean seeds stored for 18 months in a non-climatized than in a climatized environment.

### 4.2 Changes in the apparent specific mass of stored soybean seeds

The storage environments influenced the increase in the respiration of the seeds, resulting in a high consumption of dry matter and reducing the apparent specific mass of the seeds during storage. Storage in natural environment conditions reduced the specific mass of soybean seeds in the first months, which remained below that obtained from other storage conditions and was constant over time. Increases in the water content and seed mass temperature as well as in the water activity and intergranular relative humidity can result in increases in the seed respiration rates and, consequently, in higher CO_2_ concentrations, thereby resulting in greater loss of matter drought and the deterioration and reduction of the apparent specific mass of seeds stored over time [27–31].

### 4.3 Storage effects on the germination of soybean seeds

According to Hartmann Filho et al. [18], the germination capacity of soybean seeds stored for six months in a non-conditioned environment is above 80%, which is considered the minimum standard for commercial soybean seeds. However, the performance of soybean cultivars under uncontrolled conditions reduced in a germination test after three months of storage, with greater performance reductions after six months [32].

Non-climatized environments were inefficient in preserving the quality of soybean seeds during storage. According to Neve et al. [33], the performance of soybean seeds stored for six months in a non-conditioned warehouse is reduced. Carvalho et al. [34] evaluated soybean seeds stored in an uncontrolled environment for eight months and observed a reduction in their germination performance after the fourth month of storage.

Zuffo et al. [20] discovered losses, below the 80% minimum quality standard that is required for commercial seeds, in soybean seeds stored in a non-climatized environment for eight months. According to Conceição et al. [21], soybean seeds stored in a non-conditioned environment with a 92% germination rate, showed had 85%, 69%, and 55% germination rates after being stored for four, six and nine months, respectively. According to Carvalho et al. [32], the germination capacity of soybean seeds stored for seven months in a non-conditioned environment decreases below the commercial standard. Storage in climatized environments at lower temperatures is a favorable alternative for preserving seed quality. According to Zuchi et al. [8], refrigeration is beneficial for stored soybean seeds, as it improves their germination performance. Soybean seeds stored for six months preserve the same germination pattern when stored at 20°C; however, at 27°C, their germination rate decreases and falls below the commercial standard rate [19].

Soybean seeds with a 94% germination rate stored for seven and a half months in an air-conditioned environment at 20°C reach a 91% germination rate, while in an non-conditioned environment they reach an 84% germination rate [15]. Soybean seeds stored for eight months perform superiorly in terms of their germination rate in an air-conditioned environment at 10°C than seeds that are kept at ambient temperature [32]. High storage temperatures were detrimental to preserving the quality of soybean seeds. In a study conducted by Sarath et al. [35] on peanut seeds, the authors verified a 96% germination rate after five months of storage and an 83% germination rate in seeds stored in an uncontrolled environment.

Paraginski et al. [36] who studied corn seeds stored for twelve months, observed lower decreases in the germination percentage of seeds stored at 5°C and 15°C than in that of seeds stored at 25°C and 35°C. Bessa et al. [37] stored crambe seeds and obtained better germination rates in PET packaging, compared to laminated and high density polyethylene packaging in an air-conditioned environment at 10°C and in a non-conditioned environment [38]. Seed deterioration is a natural process and seeds are prone to losing vigor more quickly when they are stored in environments with elevated temperatures than in refrigerated environments. Likewise, Smaniotto et al. [19] reported a reduction in the quality of soybean seeds stored for six months under a high temperature (27°C), as the germination rates decreased dramatically, even for seeds with low water content, owing to the direct influence of storage time and temperature.

### 4.4 Changes in the electrical conductivity of stored soybean seeds

According to Neve et al. [33], the electrical conductivity of many soybean seeds that have high vigor is below 80 μS cm^-1^ g^-1^, while these values may vary depending on the cultivar. Thus, Zuchi et al. [8] found that soybean seeds stored in a refrigerated environment have lower electrical conductivity than seeds stored in a non-conditioned environment, thus indicating a better organization of the cellular tissues of the former seeds. In bean seed evaluations, Zucarelli et al. [29] detected deterioration when they obtained higher electrical conductivity values in seeds stored in a non-refrigerated environment (58.56 μS cm^-^^1^ g^-^^1^) than in those stored in an air-conditioned environment (55.90 μS cm^-^^1^ g^-^^1^) for forty five months.

Additionally, seeds stored for six months in a refrigerated environment at 20°C had more favorable electrical conductivity results than seeds stored at ambient temperature [19]. Storing soybean seeds in environments with ambient temperatures accelerates their deterioration rate over the storage period. Virgolino et al. [23] obtained lower electrical conductivity values for chilled seeds stored in kraft paper packaging than for seeds stored in uncooled conditions in big bags.

Paraginski et al. [36] and Coradi et al. [39] found that corn seeds stored in a refrigerated environment had increased electrical conductivity values; however, the electrical conductivity values of seeds stored at ambient temperature doubled and, consequently, they suffered greater deterioration. The longer the storage time and the higher the packaging permeability, the greater the seed deterioration of the seeds. Carvalho et al. [34] observed that after six months of storage, soybean seeds had higher electrical conductivity values over time. While Carvalho et al. [32] who evaluated soybean seeds stored for seven months in a non-refrigerated environment, obtained higher electrical conductivity values and reduced seed quality at the end of the storage time.

### 4.5 Influence on the first germination count (vigor) of stored soybean seeds

The seeds that were stored in artificially refrigerated environments deteriorated less; these results verified that the temperature of the seed mass became uniform faster, thereby reducing the water vapor exchanges and the effect of the heat sources and regulating the humidity that resulted in a hygroscopic equilibrium condition that was favorable for storage. According to Berbert et al. [40] and Zhang et al. [41], water content is the most significant factor that should be controlled in order to prevent seed deterioration during storage.

Smaniotto et al. [19] found that the initial water content influenced the quality of soybean seeds during storage, and seeds stored with a higher initial water content of 14% (w.b.) suffered greater quality loss during storage. Smaniotto et al. [19], who studied the biochemical changes and the physiological potential of soybean seeds, observed that storing seeds under adverse conditions reduced their physiological potential and damaged their structure; additionally, soybean seeds harvested at different times and with high physiological potential showed differences in their izoenzymatic patterns over the storage period.

There are no significant variations in the results of the first germination count of soybean seeds stored at ambient temperature for three months [23]. However, Carvalho et al. [34] observed a reduction in the germination rate and the first germination count of soybean seeds stored for eight months differing only between the storage times. No reductions have been observed in the first germination count of soybean seeds stored for two months in a non-climatized environment [42]. However, after six months of storage, such a reduction occurred owing to the increased deterioration of soybean seeds, especially in these stored at ambient temperature.

Cardoso et al. [22] observed that crambe seeds stored for six months in metallic packaging performed better in terms of the first germination count; these results were not different from those obtained from seeds stored in PET packaging. According to Sarath et al. [35], the first germination count of peanut seeds stored at ambient temperature for five months decreased from 94% to 63%. After twelve months of storage, the best first germination count results were obtained from carioca bean seeds stored in an air-conditioned environment at 20°C [26].

### 4.6 Mechanical damage from moisture in stored soybean seeds

According to Afonso Junior et al. [43], the high water activity incurred by the storage environment causes an increase in the respiratory rate of seeds and an increase in their water content, thereby leading to an increase in the metabolic rate and the temperature of the seed mass. According to Carvalho and Nakagawa [44,45], the seed coat is the main water absorber, which, when subjected to different temperature, time, and storage package conditions, influences the water level variations in the respiratory process, in metabolic activities, and in seed germination, thereby increasing mechanical damage.

### 4.7 Soybean seed vigor based on the results of the tetrazolium test

The germination and vigor test results of Forti et al. [46] suggest that uncontrolled storage environments cause a greater reduction in the physiological potential of soybean seeds compared to the dry (50% RH and 20°C) and cold (90% RH and 10°C) chambers. According to Neve et al. [33], soybean seeds stored for six months in a non-air-conditioned warehouse lose their vigor and viability. On the other hand, Ferreira et al. [15] observed that soybean seeds stored for seven and a half months in an air-conditioned environment at 20°C preserved their vigor and viability better compared to seeds stored in a non-conditioned environment with the use of the tetrazolium test.

Ferreira et al. [15] observed that soybean seeds kept under cold storage at 20°C had better vigor than uncooled seeds or seeds cooled to 17°C and stored in uncooled environments for seven and a half months. Zuchi et al. [8] found that there was no significant difference in terms of vigor and viability, according to the tetrazolium test results, between chilled and non-chilled soybean seed batches stored for four months.

### 4.8 Soybean seed viability the tetrazolium test

The use of soybean seeds with high physical, genetic, physiological, and sanitary quality standards is the main contributing factor in the successful establishment of cultures in the field [47–50]. Mechanical damages seriously impair soybean seed quality, and they can influence negatively the viability and vigor of the seeds during storage [47–49]. According to Cunha et al. [51], soybean seeds lose their viability after six months of storage in tropical conditions, but in artificial cooling conditions, they can preserve their physiological quality during twelve months of storage.

Demito and Afonso [11] found that seeds cooled artificially from 15°C to 12°C preserve their germinative power during five months of storage. However, owing to the morphological arrangement of soybean seeds that provides little protection to the embryonic axis, as this is surrounded by a thin coat [52,53], the seeds become more susceptible to mechanical damage which is considered an important cause of decreased seed quality.

### 4.9 Cluster and main component analyses of the environments, storage time, and soybean cultivars

According to Zuffo et al. [20], the longer the storage time, the lower the seed quality. The storekeeper can mitigate the process of seed deterioration only by controlling the biotic and abiotic factors. The soybean seeds that were stored in a non-conditioned environment for eight months, had reduced water content, germination, total dry mass, electrical conductivity, vigor, and tetrazolium viability. Virgolino et al. [23] observed that artificial cooling, ambient temperature, and different types of packaging had similar effects on the physiological quality of seeds. However, “big bag packaging” being the raffia bag with a capacity of one ton of seeds is more efficient in preserving the water content of chilled seeds, while the authors did not observe any direct effects of cooling on the germination and the vigor of soybean seeds.

Filho [54] stated that ambient temperature and relative humidity are the main factors that preserve seed quality during storage. Seeds stored in high temperatures and with high water content have high respiratory rates, which accelerate their deterioration by speeding up the consumption of their reserves, generating physiological wear, and decreasing their germination rates and their vigor [55,56]. Rosa et al. [57] observed that the physiological quality of seeds was superior in cold storage compared to storage under ambient conditions; additionally, the lots of cultivars FPS Jupiter, FPS Urano, FPS Antares, FPS Neptune, and CD 250 showed high twinning and vigor, with variations in their germination results, germination speed index, first count of the germination test, and electrical conductivity. Demito and Afonso [11] observed that the physiological quality of artificially cooled soybean seeds was preserved over five months of storage and they had a higher germination percentage than uncooled seeds.

According to Villela and Menezes [9] the main objectives of storage are to preserve the viability and vigor of the seeds. According to the authors, the associations between temperature and humidity influence seed longevity during storage and every 5.5°C temperature decrease during this stage may double seed longevity, while also allowing a reduction in the consumption of seed reserves by pathogens or by your own breathing process. Carvalho et al. [32] reported that the aim of current research is to facilitate the selection of soybean genotypes whose seeds have greater storage capacity; however, there are currently few studies that examine the effect of genetic diversity on the physiological quality of soybean seeds in the post-harvest period.

Mengarda et al. [58] who tested parental genotypes, identified some with higher performance in terms of seed quality. Gris et al. [59] also found dissimilarities in relation to the physiological quality of seeds. According to VanUtrecht et al. [60], seeds subjected to unfavorable conditions suffer physiological damages that impair the performance and quality of the seed lot, at different intensity levels, owing to the genetic and intrinsic factors of each cultivar.

## 5. Conclusions

The seeds of the M-SOY 8866, M7110 IPRO, CD 2737 RR, and BMX DESAFIO 8473 RSF soybean cultivars performed better in the physiological quality tests conducted in different storage environments.

The storage duration had a cumulative effect on the negative factors that favor the deterioration of the quality of the stored seeds.

The storage temperature was the main factor that affected the physiological quality of the stored seeds. Seed storage at ambient temperature resulted in the worst physiological quality of the seeds, especially in the last month of storage.

The use of coated packaging was beneficial in preserving the physiological quality of stored soybean seeds; however, its effect was greater in ambient temperature conditions than in cold environments.

The best storage environment for preserving the physiological quality of the seeds was at 10°C with the use of coated packaging, while the worst was at ambient temperature without the use of coated packaging.

It was concluded that the use of coatings in raffia big bags can be an alternative for maintaining the quality of seeds of different soybean cultivars during storage in seed processing units.

## Supporting information

S1 Table Data set as a supporting information. (XLS)
